# Structural Dynamics of Lytic Polysaccharide Monooxygenase during Catalysis

**DOI:** 10.3390/biom10020242

**Published:** 2020-02-05

**Authors:** Frantisek Filandr, Daniel Kavan, Daniel Kracher, Christophe V.F.P. Laurent, Roland Ludwig, Petr Man, Petr Halada

**Affiliations:** 1 Institute of Microbiology of the CAS, Division BioCeV, Prumyslova 595, 252 50 Vestec, Czech Republic; frantisek.filandr@biomed.cas.cz (F.F.); kavan@biomed.cas.cz (D.K.); 2 Department of Biochemistry, Faculty of Science, Charles University, Hlavova 2030/8, 128 43 Prague 2, Czech Republic; 3 Biocatalysis and Biosensing Research Group, Department of Food Science and Technology, BOKU—University of Natural Resources and Life Sciences, Muthgasse 18, 1190 Vienna, Austria; danielkracher@boku.ac.at (D.K.); roland.ludwig@boku.ac.at (R.L.)

**Keywords:** lytic polysaccharide monooxygenase, lignocellulose degradation, hydrogen/deuterium exchange mass spectrometry, oxidative amino acid modification, peptide bond cleavage, reactive oxygen species

## Abstract

Lytic polysaccharide monooxygenases (LPMOs) are industrially important oxidoreductases employed in lignocellulose saccharification. Using advanced time-resolved mass spectrometric techniques, we elucidated the structural determinants for substrate-mediated stabilization of the fungal LPMO9C from *Neurospora crassa* during catalysis. LPMOs require a reduction in the active-site copper for catalytic activity. We show that copper reduction in *Nc*LPMO9C leads to structural rearrangements and compaction around the active site. However, longer exposure to the reducing agent ascorbic acid also initiated an uncoupling reaction of the bound oxygen species, leading to oxidative damage, partial unfolding, and even fragmentation of *Nc*LPMO9C. Interestingly, no changes in the hydrogen/deuterium exchange rate were detected upon incubation of oxidized or reduced LPMO with crystalline cellulose, indicating that the LPMO-substrate interactions are mainly side-chain mediated and neither affect intraprotein hydrogen bonding nor induce significant shielding of the protein surface. On the other hand, we observed a protective effect of the substrate, which slowed down the autooxidative damage induced by the uncoupling reaction. These observations further complement the picture of structural changes during LPMO catalysis.

## 1. Introduction

Plant-biomass is a major source of renewable energy in the form of biofuels and bio-produced chemicals, but the recalcitrance of lignocellulose is a major obstacle to cost-effective saccharification. Fungal copper-dependent lytic polysaccharide monooxygenases (LPMOs, EC 1.14.99.53, - 56) have been found to boost the overall effectiveness of lignocellulolytic secretomes in the decomposition of insoluble recalcitrant polysaccharide structures [1]. Initially described in bacteria in 2010 [2], LPMOs have since been identified in fungi and insects and have been classified by the curators of the carbohydrate-active enzyme (CAZy) database (www.cazy.org) into several auxiliary activity (AA) families [3]. Fungal LPMOs have so far been classified as AA9 (activity on soluble and insoluble beta-glucans), AA11 (chitin-active), AA13 (starch-active) [4], AA14 (xylan-active) [5], AA15, and AA16 [6]. LPMOs differ from common endo- and exo-glycosyl hydrolases by employing an oxidative mechanism to cleave glycosidic bonds in polysaccharides, such as cellulose, hemicellulose, starch or chitin, to produce access points for exo-acting hydrolases. After prolonged incubation, they also release soluble oligosaccharide products [7,8,9,10]. The ability to cleave polymers is enabled by a characteristic, flat binding-site consisting of aromatic and hydrophilic amino acids for the interaction with sugar moieties. An exposed active-site copper is held in place by a conserved, “histidine brace” motif [11,12,13]. These structural features enable LPMOs to attack polysaccharide surfaces inaccessible to hydrolases [13,14].

The catalytic mechanism of LPMOs is still under investigation, but several cornerstones of LPMO catalysis have been elucidated. The first step in the catalytic cycle is the reduction of the Cu(II) in the catalytic site to Cu(I), either by small molecular reductants [15,16,17], such as ascorbic acid (Asc) or gallic acid or by the fungal redox enzyme cellobiose dehydrogenase (CDH) [18]. CDH most likely requires the dissociation of LPMO from the substrate in order to contact the otherwise buried copper center of LPMO [19]. The reduction of the copper ion is also accompanied by a conformational change in LPMO, as was observed by using NMR [20] or electronic circular dichroism [21], and it also increases the affinity to its carbohydrate substrate [21,22]. As co-substrate for the LPMO reaction, either O_2_ [23] or H_2_O_2_ [24] have been suggested. Whether the binding and reduction in an oxygen species happen preferentially in the free or substrate-bound state is not settled, but a recent publication suggests that hydrogen peroxide can access the active side of substrate-bound LPMO [25]. The catalytically competent LPMO abstracts hydrogens from its polysaccharide substrate and thereby breaks the glycosidic bond [23,26,27]. Oxidation on both C1 and C4 positions of the polysaccharide substrate was reported, with different resulting products. C1 oxidation leads to the formation of a lactone which spontaneously hydrolyzes into gluconic acid, while C4 oxidation forms a C4 ketone that hydrolyzes into a gemdiol in aqueous solution [7,27,28]. Several studies have shown that H_2_O_2_ increased the reaction rate relative to O_2_ by an order of magnitude [24,29,30] while the measured affinity of LPMO for H_2_O_2_ was in the micromolar range [24,29]. The proposed reaction mechanism based on H_2_O_2_ as cosubstrate would also require only one electron to initiate the catalytic reaction, which aligns well with CDH’s capability of transferring single electrons via its cytochrome domain. Several studies reported that the presence of H_2_O_2_-scavengers, such as peroxidase or catalase, inhibited the LPMO reaction, which implies that H_2_O_2_ is required for the LPMO reaction [21,24,30]. By providing a mix of different H_2_O_2_ and O_2_ isotopes, Bissaro et al. showed that the oxygen atom inserted into the LPMO reaction products originated from the H_2_O_2_ [24]. A recent paper showed that potentially both O_2_ and H_2_O_2_ can serve as co-substrates for LPMO, resulting in polysaccharide oxidative cleavage, but concluded that different molecular mechanisms are probably employed [30].

LPMOs were reported to be notoriously unstable under non-optimal reaction conditions. Especially, the observed higher catalytic rates in the presence of H_2_O_2_ were accompanied by a rapid inactivation of LPMO [30]. Employing LPMO in a commercial cellulase cocktail using industrially relevant substrates, therefore, requires fine-tuning of the reaction rate by controlling the H_2_O_2_ supply [31]. The observed instability was mainly attributed to autoxidative damage caused by oxygen radicals released from the active site, which lead to the oxidation of amino acids surrounding and forming the active site of LPMO [21,24,32]. The apparent T_m_ of the fungal LPMO9C from *Neurospora crassa* decreased from 61.5 ± 0.4 °C to 48.8 ± 1.1 °C when the active site copper was reduced by Asc. Under these conditions, H_2_O_2_ accumulates via the oxidation of O_2_ by Asc [33,34]. In addition, the reduced LPMO may also generate low amounts of H_2_O_2_ through an uncoupling reaction [35]. In the presence of suitable substrates, however, the apparent T_m_ of *Nc*LPMO9C remained relatively unchanged at 60.4 ± 0.5 °C [21]. The stabilization of reduced LPMO by the substrate can be explained by the prevention of freely diffusing oxygen radicals due to the catalytic reaction [36], which may also consume the generated H_2_O_2_.

In addition to autooxidative damage, another form of destabilization was observed upon removal of the active site copper, e.g., by incubation of LPMO with an excess of the metal chelator - ethylenediaminetetraacetic acid (EDTA) [21,37]. Several studies showed reduced temperature stability of copper-depleted LPMOs [21,38]. For example, removal of copper reduced the apparent T_m_ of *Nc*LPMO9C from 61.5 to 52.7 °C [21], which indicates that the histidine brace motif and the presence of the copper ion stabilize the overall protein fold. The thermal stability of the apoenzyme was unaffected by the presence of reducing agents and substrates, showing that an intact active site is required for substrate recognition and catalysis. While it is possible that the loss of the active-site copper ion contributes to the initial LPMO destabilization following active-site reduction and subsequent oxidation, it was demonstrated that the vast majority of LPMO molecules retained the copper ion upon unfolding.

In this work, we study the destabilizing effect of reducing agent and copper ion removal on *Nc*LPMO9C in temporal and structural detail by hydrogen/deuterium exchange mass spectrometry (HDX-MS). We also investigate the stabilizing effect of carbohydrate substrates during catalysis. The employed HDX-MS methods are well suited for the detection of structural changes involving the rearrangement of hydrogen bonds and changes in solvent accessibility in proteins, and they allow studying heterogeneous reactions with insoluble components, as long as they can be quickly removed before MS analysis. This is crucial for studying LPMOs, as their typical natural substrates, e.g., cellulose, are inherently insoluble. We observed a significant increase in peptide solvent accessibility throughout the LPMO molecule when incubated with a reducing agent, starting at peptides close to the copper ion active site and then propagating further. This effect was found to be slowed down in the presence of cellulose. Removal of the active site copper ion caused a temperature-induced unfolding beginning at lower temperatures, which affected the histidine brace peptides first and then propagated to the rest of the molecule upon longer incubation times.

Thus far, amino acid modifications resulting from autooxidative damage of LPMOs have only been reported in bacterial LPMOs [24]. We, therefore, aimed to verify that the same modifications occur in fungal LPMOs. Using LC-MS/MS analysis, we show that various oxidative alterations of peptides located around the copper active site occurred in a fungal LPMO. Oxidative peptide bond cleavages and, thus, direct degradation of the protein was detected for the first time in a fungal LPMO. The peptide signal intensity observed in our HDX-MS experiments provides a measure of the given peptide abundance. We observed a decrease in the intensity for unmodified peptides located in the vicinity of the active-site, indicating their cleavage or modification.

## 2. Materials and Methods

### 2.1. Protein Samples

Expression and purification of *Neurospora crassa* LPMO9C (*Nc*LPMO9C) were performed according to a published protocol [35]. NcLPMO9C was recombinantly expressed in *Pichia pastoris* X-33 cells under control of the methanol-dependent alcohol oxidase (AOX) promoter and chromatographically purified to homogeneity. Specific activity of 5.50 U g^−1^ was determined by using the Amplex Red assay [35], with the total protein concentration being determined by the Bradford protein assay. This value is close to the reported specific activity of *Nc*LPMO9C of 5.57 U g^−1^ [35].

### 2.2. Hydrogen/Deuterium Exchange Mass Spectrometry (HDX-MS)

*Nc*LPMO9C (“holo”, 2 µM) or *Nc*LPMO9C copper-depleted by overnight incubation with 10 mM EDTA (“apo”, 2 µM) was pre-incubated at 35, 50, or 65 °C in 50 mM sodium acetate buffer, pH 6.0. Dilution into D2O deuteration buffer (50 mM sodium acetate, pD 6.0) was done 10-fold, and aliquots containing 100 pmol of *Nc*LPMO9C were removed at different time points. Deuteration of *Nc*LPMO9C alone or in the presence of 5 mM Asc, 4 mg mL^−1^ microcrystalline cellulose, or both Asc and microcrystalline cellulose was performed. These additives were added to the deuteration buffer prior to mixing with the LPMO sample. The microcrystalline cellulose was centrifuged and washed several times with a deuterated buffer to remove H_2_O and soluble oligosaccharides prior to use. Deuterium exchange in aliquoted samples was immediately stopped by mixing the sample 1:1 with quenching solution (200 mM tris (2-carboxyethyl) phosphine (TCEP), 8 M urea, 1 M glycine, pH 2.3). The sample was subsequently flash-filtered for 20 s using spin filter tubes (Ultrafree-MC GV, polyvinylidene fluoride (PVDF) 0.22 µm, Merck, Darmstadt, Germany) to remove insoluble cellulose fibers and then rapidly frozen in liquid nitrogen. Sampling, filtering, and freezing took precisely 90 s for every sample. For the peptide mapping of non-deuterated control samples, the same protocol was used with the difference of using H_2_O-based buffer instead of a D_2_O-based buffer.

### 2.3. HPLC/ESI-FT-ICR-MS Analysis of HDX Samples

Samples were stored at −80 °C, quickly thawed before the LC-MS analysis, and then injected into an in-house build LC system maintained at a stable temperature of 0 °C to minimize the deuterium back-exchange. The digestion took place on columns with immobilized proteases (rhizopuspepsin, nepenthesin I, made in-house [39,40]) using 0.4% formic acid (FA) in water as an eluent at a flow-rate of 200 µL min^−1^ (LC-20AD HPLC pump, Shimadzu, Tokyo, Japan). The resulting peptides were subsequently trapped and desalted on a peptide microtrap (Optimize Technologies, Oregon City, OR, USA). The whole digestion and desalting procedure took precisely 3 min. Desalted peptides were eluted with an acetonitrile gradient (HPLC system Agilent 1200, Agilent Technologies, Waldbronn, Germany) on a reversed-phase analytical column (ZORBAX 300SB-C18, 0.5 × 35 mm, 3.5 µm, Agilent Technologies, Waldbronn, Germany) where they were further separated. The eluting gradient from 5–35% B lasted for 5 min and was followed by a quick change to 95% B and subsequent column re-equilibration. Solvents were A: 0.4% FA, 2% acetonitrile (ACN) in water, and B: 0.4% FA, 95% ACN in water. The flow through the column was kept at 20 µL min^−1^. The column was interfaced with an electrospray ionization (ESI) source of the Fourier transform ion cyclotron resonance mass spectrometer (15T-solariX XR FT-ICR, Bruker Daltonics, Bremen, Germany). Peptide mapping was performed in a positive data-dependent MS/MS broadband mode, where each MS scan was followed by six MS/MS scans of the most abundant peptides found in the previous MS scan, which underwent collision-induced fragmentation. Deuterated samples were analyzed in the LC-MS mode.

### 2.4. Oxidative Modification Analysis

*Nc*LPMO9C was incubated at 50 °C in 50 mM sodium acetate buffer, pH 6.0, alone, or in combination with 5 mM Asc. Samples were taken in 10-min intervals for up to 30 min. The reaction was stopped by the addition of EDTA (10 mM final concentration) to chelate LPMO’s copper ion. Methionine (25 mM final concentration) was added to scavenge existing ROS. Protein samples were digested using trypsin or AspN proteases after pH adjustment to 8.8 with TRIS buffer (80 mM final concentration). Trypsin was added in a 1:100 ratio (protease:LPMO; *w*:*w*), AspN was added in a 1:300 ratio (protease:LPMO; *w*:*w*). Disulfide bonds in the samples were reduced by a 30-min incubation with 5 mM TCEP and alkylated by a 30 min incubation with 10 mM iodoacetamide in the dark at 22 °C. The pH of the samples was adjusted to acidic pH (< 2) by adding 4% trifluoroacetic acid (TFA) to stop the protease reaction. Subsequently, the samples were analyzed using HPLC/ESI-FT-ICR-MS using binding and elution solutions A and B (A: 0.1% FA, 2% ACN in water, B: 0.1% FA, 95% ACN in water) at a constant flow rate of 10 µL min^−1^, with online desalting on a reversed-phase trap column (ZORBAX 300SB-C18 5 μm, 0.3 × 5 mm, Agilent Technologies, Santa Clara, CA, USA), separation on an analytical column (ZORBAX 300SB-C18, 0.3 × 150 mm, 3.5 µm, Agilent Technologies, Santa Clara, CA, USA ) and elution during a 15 min linear gradient to 25% solution B.

### 2.5. Data Processing

Obtained LC-MS/MS data were processed by DataAnalysis 4.1 (Bruker Daltonics, Billerica, MA, USA) and then searched by MASCOT (Matrix Science, London, UK) in ProteinScape 4 (Bruker Daltonics, Billerica, MA, USA) against a database containing the *Nc*LPMO9C sequence as well as sequences of rhizopuspepsin and nepenthesin-1 as false positives/controls. Data processing combined approaches described previously [41,42]. For HDX-MS peptide mapping the no-enzyme search with no modification included was performed. Two search rounds were done—one with precursor and fragment accuracies 3 ppm and 0.05 Da, respectively. Another one, where the parent ion mass accuracy window was wider—1000 ppm and the results with errors above 3 ppm were discarded. Hits with ion scores below 20 were checked manually for fragment ion assignments and also for mass uniqueness within the *Nc*LPMO9c sequence and for isotope pattern fit. Oxidation data were also searched with multiple rounds of searches. PEAKS algorithm (Bioinformatics Solutions, Waterloo, ON, Canada) was used first to asses all possible modifications and then the data were re-searched using MASCOT. Here, the search employed small errors (3 ppm and 0.05 Da for precursor and fragments, respectively) and enzyme specificity (trypsin or Asp-N). Cys carbamidomethylation was set as fixed modification. Variable modifications were selected on the basis of PEAKS searches and included: single (+15.995) and double (+31.999) oxidation of Met, Trp, His, Tyr, Pro; His->Asp oxidative breakdown (− 22.032); oxidative peptide bond cleavage (−0.985/+25.979). All assignments were verified manually to double-check the site of oxidation. Protein purity was assessed by searching the LC-MS/MS from HDX-MS mapping and unmodified samples from oxidation analysis against NCBInr database. HDX-MS data were plotted using the DrawMap script, part of MSTools [43]. LC-MS HDX data were processed by using the in-house software DeutEx (unpublished). Deuteration levels of individual peptides at each time point were calculated. Peptides with overlapping regions were used to calculate the number of exchanged deuterium atoms to increase spatial resolution. This software was also used to calculate the peptide abundance changes as changes in MS intensity under various conditions. Data were modeled on the known crystal structure of the catalytic domain of *Nc*LPMO9C [44].

## 3. Results

A recent report from Kracher et al. described the interaction of the fungal *Nc*LPMO9C with various substrates, the enhancement of substrate binding upon active-site copper reduction, and the protective/stabilizing role of substrates on LPMO under reducing conditions. Spectroscopic techniques, such as circular dichroism, indicated conformational changes in the LPMO upon active-site copper reduction and copper depletion but did not provide insight into the structural details of the various *Nc*LPMO9C states [38,45]. In order to gain accurate structurally resolved answers underlying these phenomena, we performed a systematic hydrogen/deuterium (H/D) exchange study, which allowed us to extend the previous conclusions.

In the first step, we optimized the HDX-MS workflow, which included tuning of digestion conditions and rapid separation of proteins from the insoluble substrate during the post-labeling step. In our previous studies, which focused on cellobiose dehydrogenase, an enzyme closely cooperating with LPMO during the cellulolytic activity, we showed that strong reducing and denaturing conditions are required for efficient digestion. As these enzymes are adapted to a harsh extracellular environment, it is not surprising that LPMO digestion required denaturation. However, in contrast to the previously used guanidine hydrochloride, we switched to urea, which provided similar sequence coverage but avoided adverse effects on LC analysis. In addition, from the studies published so far, it is evident that in contrast to guanidine hydrochloride, even a high concentration of urea has much less deleterious or even enhancing effects on the protease activity [46,47,48]. We also tested various proteolytic setups (protease columns, flow rates) with serial coupling of nepenthesin-1 and rhizopuspepsin columns operated at 200 µL min^−1^, providing the best sequence coverage and spatial resolution. Finally, we had to cope with the heterogeneous, insoluble substrate (microcrystalline cellulose) present in the sample. We found the use of 0.45 µm spin filters as an optimal solution, adding approximately 50 s to the sample processing time while ensuring complete removal of insoluble matter. The harsh denaturing conditions also prevented possible adherence of LPMO to the substrate via its interaction surface and/or carbohydrate-binding module (CBM). The additional post-quench time of 90 s was also required to achieve the reduction in the disulfide bonds in *Nc*LPMO9C. We observed that raising the TCEP concentration or prolonging the incubation time did not lead to more efficient digestion and, therefore, we used the lowest possible TCEP concentration and reduction time. As a result, we fully covered the catalytic domain (except for residues 41–44) and the CBM. The linker peptide connecting the catalytic domain to the CBM (residues 225–309) could not be resolved due to its high and heterogeneous O-glycosylation (Figure S1).

**Figure 1:**
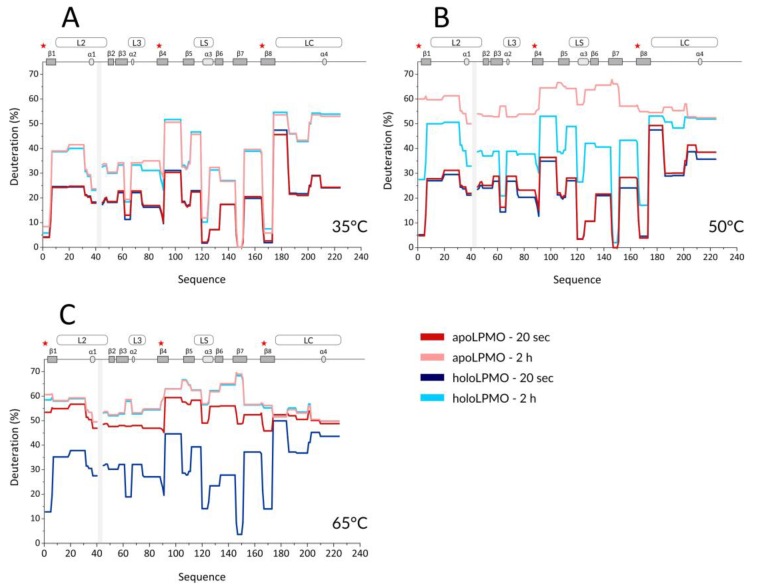
Selected hydrogen/deuterium (H/D) exchange kinetic profiles of holo- and apo-*Nc*LPMO9C at three different temperatures. Protection plots showing deuteration of *Nc*LPMO9C apo- (red tones) and holoenzyme (blue tones) at (**A**) 35 °C, (**B**) 50 °C, and (**C**) 65 °C and at two exchange times of 20 s (dark colors) and 2 h (light colors). Secondary structure elements and positions of loops are shown on the top of each graph. Shaded boxes illustrate gaps in the sequence coverage. The visualization covers the catalytic domain and part of the linker connecting the catalytic domain to the CBM. Asterisk denotes positions of the histidine brace residues (His1, His88, and Tyr166).

With the optimized workflow, we performed an initial set of HDX-MS measurements in which we focused on several factors. First, building upon the data from Kracher et al., we looked at the effect of high temperature on the free, oxidized LPMO and its apo form prepared by EDTA treatment. Here, the exchange was followed at 35, 50, and 65 °C, and the HDX kinetics covered time points at 0.33, 2, 20, 120, and 360 min. A summary of selected HDX data is provided in the protection plots in Figure 1. While this presentation focuses on the catalytic domain and representative exchange times only, we provide full data covering the whole LPMO sequence, including the CBM and all exchange times in the form of mirror plots (Figure S2) [43]. Peptides in mirror plots are represented on the *X*-axis by their “midpoint” value, which is calculated as an average value between the N- and C- terminal residue sequence position of a given peptide and thus allows for easy and reproducible sorting of the overlapping peptides generated and analyzed in the HDX-MS experiment.

**Figure 2:**
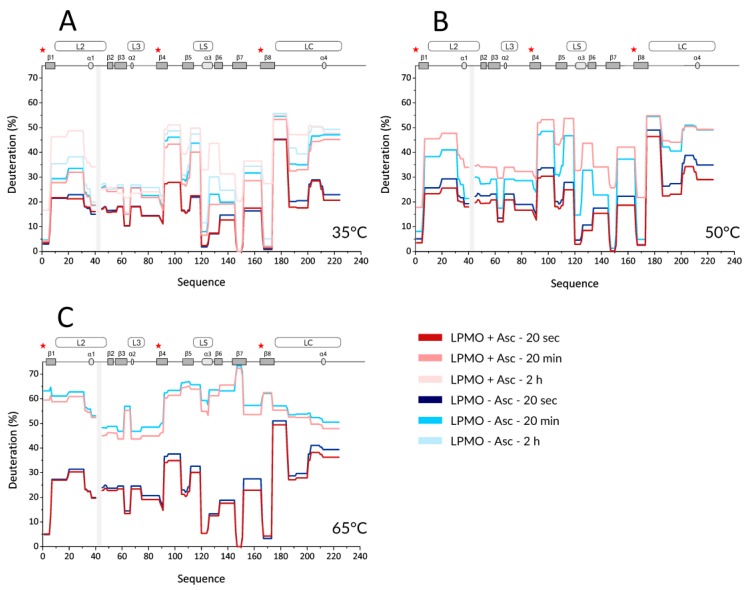
Selected H/D exchange kinetics profiles of oxidized and Asc-reduced *Nc*LPMO9C at three different temperatures. Protection plots showing deuteration of the Asc-reduced (red) and the oxidized (blue) form of *Nc*LPMO9C at (**A**) 35 °C, (**B**) 50 °C, and (**C**) 65 °C. Three exchange times of 20 s (dark colors), 20 min (lighter colors), and 2 h (the lightest colors) are shown for 35 °C (**A**), whereas (**B**) and (**C**) display only the two shorter times (20 s and 20 min, respectively). Secondary structure elements and positions of loops are shown on the top of each graph. Shaded boxes illustrate gaps in the sequence coverage. The visualization covers the catalytic domain and part of the linker. Asterisk denotes positions of the histidine brace residues (His1, His88, and Tyr166).

Since we observed no difference induced by cofactor absence or active site copper reduction at the CBM (Figure S2) and we missed a large part of the linker, we avoided these structural features in Figure 1 and Figure 2. It should be stressed that the intrinsic exchange rate was influenced by the temperature and, thus, the data acquired at different temperatures are not directly comparable. We, therefore, plotted these data separately (Figure 1A–C). However, normalization using correction factors calculated based on the Arrhenius equation can be applied to compensate for the different exchange rates. The exchange rate at 50 °C was multiplied by a factor of 3.628 and those at 65 °C by 11.739, which led to normalization to the lowest temperature of the dataset (35 °C). How this affects the data interpretation is shown in several selected deuterium uptake curves (Figure S3). From these graphs, it can be inferred that the deuterium uptake curves of the LPMO holoenzyme obtained at 35 °C and at 50 °C were either fully or partially overlapping for the whole protein and differences between these two temperatures could be seen only after longer exchange times ( > 20 min). The main differences were observed in peptides covering the histidine brace residues (His1, His88, and Tyr166) or the closely neighboring regions. This shows that even at 50 °C, the structural integrity of *Nc*LPMO9C was well preserved, and only weak destabilization occurred around the active site. In contrast, at 65 °C, the majority of the uptake curves had a much steeper slope, reaching equilibrium deuteration already after 2 min of exchange, except for unstructured and freely accessible regions (regions/peptides 92–104, 174–185, 201–224. and the CBM: 310–343). These observations clearly show that structural destabilization at 65 °C was strongly accelerated when compared with the lower temperatures. These trends were further amplified (Figure S2) when the copper was removed from the active site using EDTA treatment. When incubating the apoprotein at 35 °C, the LPMO was slightly perturbed around the active site as the deuteration increase could be observed for the peptides covering the copper-binding residues (Figure 1A and Figure S3). Interestingly, virtually no difference was detected for the region around Tyr166 (see peptides 152–165 and 166–173 in Figure S3—black and grey curves). The importance of the copper ion on the overall structure stabilization is demonstrated by a significant structure opening at elevated temperatures. At 50 °C, the apo-protein was much more accessible for deuteration (Figure 1B and Figure S3), and this was further enhanced at the highest temperature monitored (65 °C) where the protein was readily deuterated even after 20 s of deuteration (Figure 1C, Figures S2C and S3). To position these effects onto the structure, we colored the LPMO structure using the difference in HDX between the apo- and holoenzyme (Figure S4), which illustrates the destabilization of the protein core upon copper ion removal.

Next, we performed HDX experiments targeting effects induced by ascorbic acid (Asc). To allow for time-resolved analysis of the effect of LPMO reduction, we added Asc at the same time when the deuteration was started. To be able to compare the results to holo- and apo-form experiments, the reduction by Asc was also followed at three different temperatures (35, 50, and 65 °C). Key data are shown in the form of protection plots in Figure 2, while the whole dataset was visualized using mirror plots (Figure S5). The most obvious change observed at 35 °C was a generally higher deuteration of the reduced LPMO at longer incubation times (> 2 h, Figure 2A—the lightest colors). This was observed for peptides covering β1 (including the N-terminus with His1), loop L2, β2, β3-α2 linker (in L3), β5, α3 in LS, β6, β7, β8 (including Tyr166), and the middle part of loop LC. Interestingly, no such effects were detected for peptides covering the third His brace residue, His83. The protection plots showed that the peptides covering His83 were slightly less deuterated in the presence of Asc and at longer incubation times. Besides the above-described effects detected at longer time scales (> 2 h), there were also indications of lower deuteration of the reduced enzyme at earlier time points (Figure 2A-light and dark colors). However, these differences were small and cannot be considered as significant without further supporting observations. Such additional validation was obtained through inspection of data acquired at 50 °C (Figure 2B and Figure S5B). Here, the lower deuteration at 20 s and 2 min in the presence of Asc was more obvious and manifested through the peptides covering the His brace residues, including His83. Lower deuteration was also observed in neighboring peptides (e.g., L2, LS with α3, N-term part of β6, β7) or parts distant from the active site (e.g., β2, β4- β5, N-term part of LC) of *Nc*LPMO9C. On the other hand, the destabilizing effect of Asc was observed as a much higher deuteration occurring already after 20 min of exchange/reduction. Intriguingly, we observed a significant decrease in deuteration at longer incubation times beyond 2 h, which was specific for certain peptides, including the His brace residues (Figures S5 and S6A). 

We observed such unusual effects if protein degradation or precipitation occurred during the H/D experiment. Indeed, an inspection of the summed up peak intensity plots of the individual peptides (alternative data visualization in DeutEx, Figure S6B) showed a signal decrease that correlated with the trend of deuteration decrease. Hence, we conclude that at 50 °C and in the presence of Asc, LPMO is likely oxidized and/or degraded, which leads to the signal loss. At 65 °C, the effects induced by copper ion reduction were fading, and the only prominent event we observed was degradation after longer reduction/deuteration times (Figure 2C and Figure S5C). This is likely due to the destabilization of LPMO at this temperature, which we also detected for oxidized LPMO. However, considering that we still detected signal loss on peptides close to the active site even at 65 °C (see Figure S5C), we speculate that even at this temperature, the copper ion could remain bound to the His brace and the enzyme at least partially exerts its activity. To put the Asc-induced effect into a structural perspective, the difference in deuteration between reduced and oxidized *Nc*LPMO9C was plotted onto the structure (Figure S7). 

**Figure 3:**
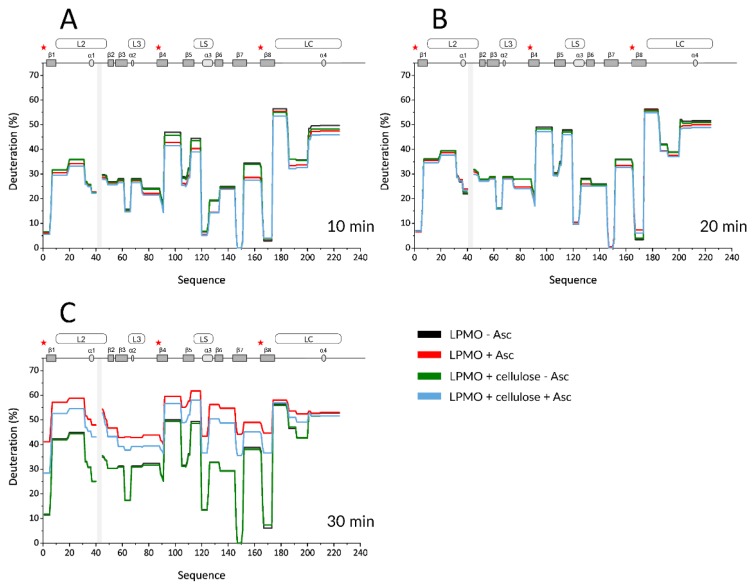
Selected H/D exchange kinetics profiles of oxidized and Asc-reduced *Nc*LPMO9C in the presence of microcrystalline cellulose. Protection plots showing deuteration profiles of oxidized (black) and Asc-reduced (red) *Nc*LPMO9C alone or in presence of cellulose (green and purple, respectively) at three deuteration times—3 min (**A**), 10 min (**B**), and 30 min (**C**). The exchange was followed at 50 °C and reduction with Asc was induced at the same time as deuteration. Secondary structure elements and positions of loops are shown on the top of each graph. Shaded boxes illustrate gaps in the sequence coverage. The visualization covers the catalytic domain and part of the linker. Asterisk denotes positions of the histidine brace residues (His1, His88, and Tyr166).

Based on these data, we then set up the final experiment. A temperature of 50 °C was chosen, as the kinetics of H/D changes were slow at 35 °C and, on the other hand, the protein was significantly perturbed at 65 °C. In addition, given the autooxidative damage detected in the experiments with Asc, the maximum exchange time was reduced to 45 min. We increased the temporal resolution of these experiments by collecting a higher number of samples and focused on the substrate binding and its protective role on the LPMO. The experimental conditions included oxidized and Asc-reduced LPMO, either alone in solution or mixed with microcrystalline cellulose. Representative HDX data are shown in the form of protection plots in Figure 3, and the whole dataset is shown as mirror plots in Figure S8. The profile of the oxidized LPMO was virtually indistinguishable from that obtained in the presence of cellulose, which implies that there is no tight binding that would alter the H/D exchange of the protein (Figure 3: black vs. green and Figure S8A). Similar to previous experiments, reduction by Asc led to two distinct effects (Figure 3—black vs. red and Figure S8B).

First, lower deuteration was detected for reduced LPMO on the peptides covering the His brace and the neighboring regions, mainly L2, L3, and its extension to β4, LS, and LC. This effect lasted until 10–15 min of deuteration. Second, the difference in deuteration between oxidized and reduced LPMO vanished after 15–20 min, and the reduced LPMO became more deuterated at later time points. This was likely linked to the oxidation-induced LPMO damage, which, after 35 min, also led to an apparent decrease in the deuteration of the His brace-covering peptides. Finally, the trends observed for free, reduced LPMO were also observed when cellulose was added. 

**Figure 4:**
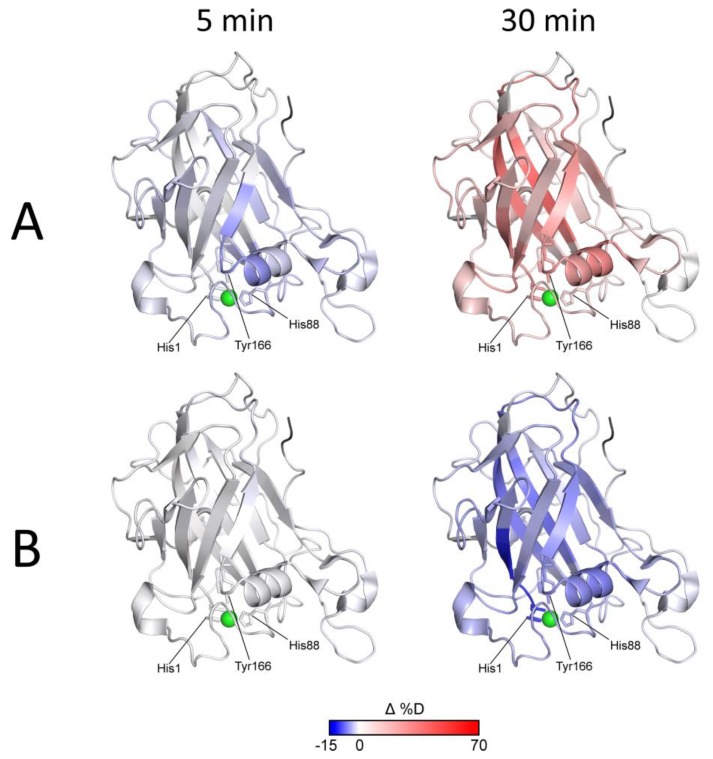
Visualization of effects exerted by Asc-reduction and cellulose binding on the structure of NcLPMO9C. Structure of NcLPMO9C (pdb: 4D7U) was colored according to the differences in deuteration. (**A**) Asc-reduction effects—deuteration of free reduced LPMO was subtracted from deuteration of the free oxidized form and the structure was colored according to these differences. (**B**) protective effect of cellulose binding/presence—deuteration levels of Asc-reduced LPMO were subtracted from the levels of LPMO reduced in the presence of cellulose. Two exchange times of 5 min and 30 min (also indicated above the structures) are shown. The full dataset covering 3 min to 35 min of incubation is shown in Figure S9. The blue-white-red gradient covers the range from –15% to 70% with white at 0%. Histidine brace residues His1, His88 and Tyr166 are labeled and their side chains are shown as sticks. Active site copper ion is shown as green ball.

Although it was shown that LPMO binds to microcrystalline cellulose under reducing conditions, albeit weakly compared to more amorphous phosphoric acid swollen cellulose (PASC) [21], we observed no decrease in deuteration in the first data points due to the presence of cellulose. However, the transition point at which the reduced protein switched from a more protected to a more deuterated state was shifted, indicating that oxidation and degradation are slowed down due to the protective role of the cellulose substrate. The last data points of the kinetics showed a deuteration decrease (described above for free reduced LPMO), which can be considered as an indicator of protein degradation. This effect was observed for both the free and cellulose-bound and Asc-reduced LPMO, which shows that the autooxidative damage at a certain point overrode the protective effect of the cellulose. We also observed protection from exchange on the CBM upon cellulose addition. This effect is not affected by the presence of Asc and justifies the binding of LPMO to the substrate. However, the extent of this protection was very small and at the border of significance. All these effects were visualized on the LPMO structure (Figure 4—selected time points and Figure S9—whole dataset, excluding oxidized LPMO conditions where no difference was detected). 

The effects of Asc reduction (protection/lower deuteration) and subsequent autooxidative damage to LPMO (higher deuteration) are evident from the blue-to-red transition shown in Figure 4A and Figure S9A. Figure 4B and Figure S9B show that the expected tighter LPMO binding to cellulose did not cause detectable alterations in the HDX kinetics and that only protection from autooxidative damage could be captured using HDX-MS. It should be noted that this protective effect aligned well with the timing of increased oxidative damage (detectable after 15 min of exchange/reduction) and was diminished after 30 min when oxidative damage prevailed. Oxidation and/or degradation and the protective effect of the cellulose were also supported by the plots following signal intensity throughout the experiment. These are shown in Figure S10 together with the respective deuterium uptake plots for the peptides covering the His brace and several representative parts of the protein structure. It is evident that in cases where the deuteration drop occurred during the last time points, the signal intensity also decreased (Figure S10A,B). The protective effect of the cellulose substrate under reducing conditions then manifested as slower deuteration, and it also slowed-down the degradation. The structural and spatial dependences of these effects could be deduced from the localization of the peptides on the *Nc*LPMO9C structure. Here, it is evident that the proximity of peptides to the catalytic center was the major factor behind the signal loss (amino acid oxidation extent, Figure S10C).

In order to verify that the mechanism underlying signal loss and deuteration decrease is indeed protein oxidation caused by reactive oxygen species generated by the reduced LPMO, we searched for oxidized versions of the peptides detected in HDX-MS. However, this approach was largely unsuccessful, with only a few significant hits. This can be demonstrated by peptide 1–6 (Figure S11) for which the singly oxidized version (oxidation of His1, part of the His brace) was found. The deuterium uptake (Figure S11A) had a different shape when compared with the unmodified peptide and increased rapidly starting from the early time-points on. In addition, the time-dependent signal intensity (Figure S11B) showed the opposite trend. While the unmodified peptide vanished, the intensity of the oxidized peptide increased, showing that the oxidation was ongoing throughout the experiment.

**Figure 5:**
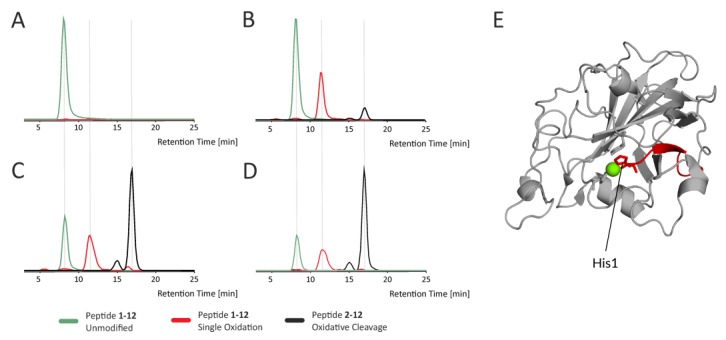
Monitoring kinetics of LPMO oxidative modifications by mass spectrometry. Extracted ion chromatograms for the N-terminal LPMO peptide (1–12) that contains His1 residue involved in copper ion binding. LPMO were incubated (**A**) alone for 30 min, or with 5 mM ascorbic acid for (**B**) 10 min, (**C**) 20 min, (**D**) 30 min. Subsequently, it was digested with Asp-N and analyzed by LC-MS/MS. Chromatographic traces show a signal for unmodified (green), oxidized (+O1; red) and oxidatively cleaved (−His, +C1O1-H2; black) peptides. The data clearly shows how the relative amount of the singly oxidized form increases, followed by oxidative cleavage, while the relative intensity of the unmodified peptide is decreasing. Localization of the peptide and the His residue is shown on a structure in (**E**). Copper ion is shown as green ball.

A detailed description of oxidative modifications in LPMO was done using a classical proteomic bottom-up approach based on the digestion of the protein with specific proteases. *Nc*LPMO9C was subjected to reduction using Asc, and protein samples were taken at different incubation times and digested using Trypsin or Asp-N proteases. The breakdown products were analyzed by LC-MS/MS, followed by a database search and manual validation. Due to the sheer amount of variability in oxidative modifications present, we only focused on the most abundant peptide forms, which yielded intense fragment spectra of sufficient quality to verify the modification type and residue position. An overview of the identified oxidative modifications is provided in Table S1**.** The analysis confirmed that similar to a previous report on *Sc*LPMOC [24], *Nc*LPMO9C underwent extensive autocatalyzed oxidation in the presence of Asc. When searching our dataset, we found not only known oxidative modifications of amino acid side chains but also generally overlooked peptide bond cleavages [49,50]. These peptide bond cleavages result in either +25,980 Da or -1,030 Da mass changes in the N-terminus of resulting shorter cleaved peptides and -30,010 Da or -0,985 Da mass change in the C-terminus, depending on the cleavage mechanism. An example shown in Figure 5 shows the extracted ion chromatograms of various forms of the N-terminal peptide bearing His1 (part of His brace), which belongs to a group of rapidly oxidized regions undergoing further oxidation-induced events, such as oxidative cleavage. 

Generally, the extent and kinetics of oxidation depended on the proximity of the peptide to the catalytic center and on the amino-acid composition of the peptide, as shown in Figure S12. It is apparent from both Figures S10 and S12 that residues close to the active site were oxidized faster than those located on the opposite side of the protein.

## 4. Discussion

Previous reports described thermal aspects of LPMO stability in relation to its cofactor and substrates [21,45]. Other publications focused on the structural differences between the apo- and the holoenzyme and substrate binding effects. These employed protein X-ray crystallography or NMR providing excellent spatial resolution and detailed answers about key amino acids and their side chains involved in these interactions. Here, we employed hydrogen/deuterium exchange, which is a lower-resolution structural biology method but offers a more detailed view on the dynamics of thermal- and ROS-induced destabilization of proteins. It also complements the crystallography and NMR data by allowing the monitoring of different conditions not easily amenable by either of the two classical techniques. We also employed hydrogen/deuterium exchange to follow the deactivation of reduced LPMO in a time-dependent manner. Thereby, we shed light on the nature of the destabilization, which is important in the context of effective industrial utilization of LPMOs.

Our first experiment aimed to elucidate the structural differences between the apo- and holo-forms of *Nc*LPMO9C and their stability at different temperatures. A previous NMR study followed the titration of the apoLPMO by Cu^2+^ and provided evidence about changes mainly at the copper coordinating residues and their surroundings [20]. Other studies based on thermal unfolding assays then showed that the absence of the copper ion results in structural unfolding occurring at lower temperatures when compared to the holo form [21,22,23,24,25,26,27,28,29,30,31,32,33,34,35,36,37,38]. Using HDX-MS, we further extended these findings by providing spatially resolved data on *Nc*LPMO9C. We selected three temperatures representing important points on the *Nc*LPMO9C unfolding pathway. At 35 °C, a very mild structure perturbation was detected (Figure 1A and Figure S4A). Slightly higher deuteration of the apo form was detected for peptides covering His1, His83, and Tyr166 after 2 h of incubation. Kinetics of deuteration varied between the individual amino acids forming the His brace. Changes in His83 manifested mostly between 2 and 20 min of deuteration, while changes in His1 and Tyr166 were observed only at longer deuteration times between 2 and 6 h (Figure S3). This is likely related to the location of these residues in the protein structure. His1 and Tyr166 are both linked to the core beta-sandwich, which could result in slower deuteration despite the fact that both amino acids are solvent-exposed. In contrast, His83 is positioned in the loop L3 connecting helices 3 and 4 and is thus less protected from an exchange. At 50 °C, the crucial role of the copper ion in the stabilization of the overall protein fold manifested (Figure 1B and Figure S4B). Detected deuteration differences were similar to those at 35 °C, albeit much more pronounced, mainly in regions covering the histidine brace residues (especially His1 and Tyr166) but also for regions 61–66, 120–125, and 146–151. These segments are either forming the beta-sandwich core (146–151) or are positioned close to the catalytic center (61–66, 120–125). Interestingly, the region 105–111, which is distant from the catalytic center, showed a high degree of perturbation as well, which suggests the overall “disassembly” of the LPMO core upon removal of the copper ion. Finally, we observed that the highest employed temperature of 65 °C leads to very rapid deuteration of both LPMO states with slower kinetics present in the holo-form (Figure 1C and Figure S3). This indicates that the copper ion was probably still bound to the enzyme, as was previously observed for fungal and bacterial LPMOs [21,38].

The function of LPMOs depends on the reduction state of their active-site metal ion. The reduction and subsequent catalytic activity, however, leads to rapid inactivation of the enzyme in the absence of suitable substrates. This inactivation was attributed to the formation of oxygen radicals and subsequent oxidative modifications of amino acid residues lining the active site [19,22,28]. Here, the accompanying structural changes were investigated through another set of HDX-MS experiments, and the oxidation pathways were further explored by LC-MS/MS analyses. Among the three temperatures followed in this experiment, 50 °C was the most informative temperature since the differences at 35 °C were only observable after long incubation times and both states (oxidized and reduced) were highly perturbed at a higher temperature of 65 °C (Figure 2 and Figure S5). Two types of changes in the Asc-reduced LPMO structure could be observed at 50 °C (Figure 2 and Figure S6). At short incubation times, we observed a low deuteration of peptides covering the histidine brace motif and of some of the loops surrounding the active site (Figure S7). This change in structure could be responsible for the increased affinity of the reduced LPMO to the polysaccharide substrate [21]. Similar loops were also found to be responsible for substrate binding of a bacterial LPMO [20] and could correspond to structural changes observed in *Nc*LPMO9C using circular dichroism [21]. A second important observation was an extensive increase in deuteration of reduced *Nc*LPMO9C at longer incubation times. Deuteration started on peptides bearing the histidine brace residues and propagated to the rest of the molecule during the incubation time. At a temperature of 50 °C, even the innermost parts of the protein were highly deuterated after 30 min, clearly showing enzyme unfolding and probably partial protein degradation. Therefore, we concluded that the optimal conditions to follow the Asc-induced reduction and LPMO substrate binding are best represented by 50 °C and shorter incubation/deuteration times, likely not exceeding 1 hr.

Based on these findings, we set up the final experiment in which the protein was followed in its oxidized and reduced state either in solution or bound to a substrate. Finer sampling clearly supported the conclusions of the previous experiment, which showed that Asc reduction (Figure 4A, Figures S9A and S10) caused a lower deuteration at shorter incubation times, transitioning towards increased deuteration at longer incubation times. Finally, we observed signal loss events in amino acids localized mostly around the histidine brace. Inclusion of microcrystalline cellulose as a substrate showed no deuteration effects under oxidizing conditions, which agrees well with the previous findings [21]. However, when the cellulose was added to the reduced LPMO, a protective effect was detected via slower deuteration. As can be seen in Figure 3C, Figures S9A and S10, the substrate failed to stop the destructive pathways at some point (time > 35min), and the oxidation-driven LPMO damage overrode the protective abilities of the cellulose. This can likely be attributed to the weaker binding of *Nc*LPMO9C to crystalline cellulose when compared to amorphous substrates, such as PASC [21]. However, it should be noted that our initial trials with PASC provided poorly reproducible results (data not shown) and, thus, we switched to crystalline cellulose. Interestingly, we failed to observe any further decrease in deuteration induced by substrate binding. This can be again attributed to the weaker binding of the LPMO to microcrystalline cellulose. However, another and more likely reason might be the inability of the H/D exchange approach to capture these interactions since they are side chain mediated, and the HDX targets back-bone amides only [51,52]. The same reasoning could explain the negligible effect of crystalline cellulose on CBM. It is known from the literature that CBM binding to cellulose surface is mediated via side chains of aromatic residues [53,54]. Since the overall fold of CBM is highly compact, it is not likely that side-chain mediated cellulose-binding may induce significant changes in CBM hydrogen bonding or solvent protection detectable by HDX-MS. Hence, while we repeatedly detected Asc-independent protection of the CBM in the presence of cellulose, the effect was at the level of insignificance. Nevertheless, our experiments clearly demonstrate that the substrate protects the enzyme from degradation and that this protective effect correlates well with the mechanism through which the enzyme is structurally perturbed in its free, Asc-reduced form (compare Figure S9A,B). This protection can be due to either scavenging of the reactive species, LPMO reoxidation, or active-site protection due to substrate binding.

An additional effect we observed throughout the experiments with Asc-reduced LPMO was a time-dependent decrease in signal intensity for peptides around the active site or nearby loops. Loss of peptide signal intensity can result from the modification of some of its amino acids, leading to mass shifts and, thus, to the vanishing of the peptide from HDX-MS analysis. Such effects were used as an indirect measure of oxidative peptide modification by Loose at al. [34]. In addition to the signal intensity loss, we here attempted to identify increases in signal intensity of oxidatively modified peptides. This approach was largely unsuccessful with the reported example (Figure S11) being the only unambiguous one. It should be stressed that looking for oxidatively modified peptides in HDX-MS data is not ideal for several reasons. First, the nonspecific proteases (here a combination of nepenthesin I and rhizopuspepsin) create many overlapping peptides that cause signal intensity splitting. Second, oxidative modifications are often heterogeneous and create a variety of subsequent reaction products adding variations to the multiple peptides covering each region. Finally, the signal intensity is further decreased due to deuteration, which usually widens the isotopic structure of the peptide ions. These combined effects make all but the most common peptide forms undetectable. We, therefore, used a separate LC-MS/MS analysis step to obtain detailed information about protein modifications occurring in Asc-treated LPMO digested by either trypsin or Asp-N. This approach also helped to verify that the introduced oxidative modifications caused the observed decrease in signal intensity. However, in contrast to results obtained with *Sc*LPMO10C by Bissaro et al., we were not able to get a comprehensive list of all modifications, since *Nc*LPMO9C was not amenable by any typical proteomic-based proteolysis approach [24]. Even the use of two different proteases (trypsin and Asp-N) failed to provide fragments of reasonable size covering the entire protein sequence or at least the catalytic unit. Instead of relying on a pure search engine scoring, we performed manual validation of the MS/MS assignments and listed only those modifications where the exact oxidation position could be deciphered. This may also lead to a shortening of the list of modified residues. However, even with a less comprehensive listing, we provide clear proof that the *Nc*LPMO9C is extensively oxidized on many sites (Table S1). Besides the oxidations and the known subsequent products of the oxidation pathways, we also found prominent oxidation leading to peptide bond cleavages. These were mainly located on amino acids close to the active site (Table S1). These peptide cleavages have been identified before in other proteins [32,33], but are not very well-known and have not been connected to LPMO oxidative damage so far. Using extracted ion chromatograms from our LC-MS/MS data, we also followed the abundance of oxidatively modified peptides during incubation of LPMO with ascorbic acid (Figure 5 and Figure S12). These data show a clear increase in the level of protein oxidation over time, resulting also in peptide bond cleavages, as illustrated by peptide H1-A12 in Figure 5. In addition, there is a clear dependency between the level of oxidation at particular sites and their proximity to the active site. We highlight this in Figure S12, where side chains containing amino acids prone to oxidation are shown as sticks. Considering their surface accessibility and the rate of oxidation, which can be inferred from the extracted ion chromatograms, it is clear that the driving factor affecting the extent of oxidation in Asc-reduced LPMO is the proximity to the active center, specifically the involvement in copper ion binding (compare peptides. 94–109 and 22–45 versus 58–84).

## 5. Conclusions

Our results describe the degradation of LPMO in structural detail and confirm that the increase in stability of LPMO caused by the presence of substrate is based on the decrease in oxidative damage incurred to the enzyme over time. The developed methodological setup marks the way towards more systematic structural studies, in which the stabilizing or destabilizing effects of larger sets of substrates, reductants, and cosubstrates can be effectively probed. Such studies may help to guide the rational design of cellulose-active enzymes leading to more efficient substrate binding to increase the lifetime of enzymes used in industrial settings.

## Supplementary Materials

The following are available online at https://www.mdpi.com/2218-273X/10/2/242/s1. Figure S1: Peptide map showing the sequence coverage of *Nc*LPMO9C after serial nepenthesin-1 / rhizopuspepsin digestion, Figure S2: *Nc*LPMO9C structural changes induced by the temperature, Figure S3: Selected examples of deuterium uptake curves for holo-/apo- *Nc*LPMO9C after temperature correction, Figure S4: Destabilization of the *Nc*LPMO9C by active site copper removal as observed by HDX-MS comparison of holo- and apoenzymes, Figure S5: *Nc*LPMO9C structural changes induced by reduction with ascorbic acid at different temperatures, Figure S6: Ascorbic acid reduction causes a range of effects in HDX kinetics and a signal loss, Figure S7: The effect of ascorbic acid-mediated copper ion reduction mapped on the structure of *Nc*LPMO9C, Figure S8: The effects of the reduction of *Nc*LPMO9C by Asc and cellulose-binding in the oxidized and Asc-reduced states, Figure S9: The effects of *Nc*LPMO9C ascorbic acid reduction and cellulose-binding of the Asc-reduced LPMO visualized on the protein structure, Figure S10: Detailed monitoring of ascorbic acid reduction and its effects on HDX kinetics and signal loss, Figure S11: Oxidation induced by copper ion reduction detected in the HDX dataset, Figure S12: Monitoring of the signal intensity increase of modified peptides. Table S1: Manually verified modifications and cleavages on amino acids in LPMO recorded by MS/MS.
