# Supplementary material for: Structural Dynamics of Lytic Polysaccharide Monooxygenase during Catalysis
Source: Biomolecules. 2020 Feb 5;10(2):242. doi: 10.3390/biom10020242 (PMC7072406; doi:10.3390/biom10020242)
Supplement: Supplementary file 1 [file biomolecules-10-00242-s001.pdf]

SUPPLEMENTARY INFORMATION

Structural investigation of lytic polysaccharide monooxygenase during catalysis

Frantisek Filandr[1,2], Daniel Kavan[1], Daniel Kracher[3], Christophe V.F.P. Laurent[3], Roland Ludwig[3], Petr Man[1*] and Petr Halada[1*]

[1] Institute of Microbiology of the CAS, Division BioCeV, Prumyslova 595, Vestec, 252 50, Czech Republic. frantisek.filandr@biomed.cas.cz (F.F.); kavan@biomed.cas.cz (D.Ka.); halada@biomed.cas.cz (P.H.)

[2] Department of Biochemistry, Faculty of Science, Charles University, Hlavova 2030/8, Prague 2, 128 43, Czech Republic

[3] Biocatalysis and Biosensing Research Group, Department of Food Science and Technology, BOKU - University of Natural Resources and Life Sciences, Muthgasse 18, 1190 Vienna, Austria. danielkracher@boku.ac.at (D.Kr.) christophe.laurent@boku.ac.at (C.V.F.P.L.); roland.ludwig@boku.ac.at (R.L.)

* Correspondence: pman@biomed.cas.cz (P.M.) and halada@biomed.cas.cz (P.H.)

Includes Figures S1-S12 and Table S1

Figure S1. Peptide map showing the sequence coverage of *Nc*LPMO9C after serial nepenthesin-1 / rhizopuspepsin digestion. Blue bars represent individual peptides. Secondary structure elements extracted from pdb file 4D7U are shown above the sequence as arrows (beta-sheet) and cylinders (alpha-helix). Names and position of loops (L2, L3, LS and LC) are also indicated. The sequence of the catalytic domain was covered fully with the exception of region 41-44. The CBM was also covered and, thus, the only missing part is represented by the unstructured and highly O-glycosylated linker.

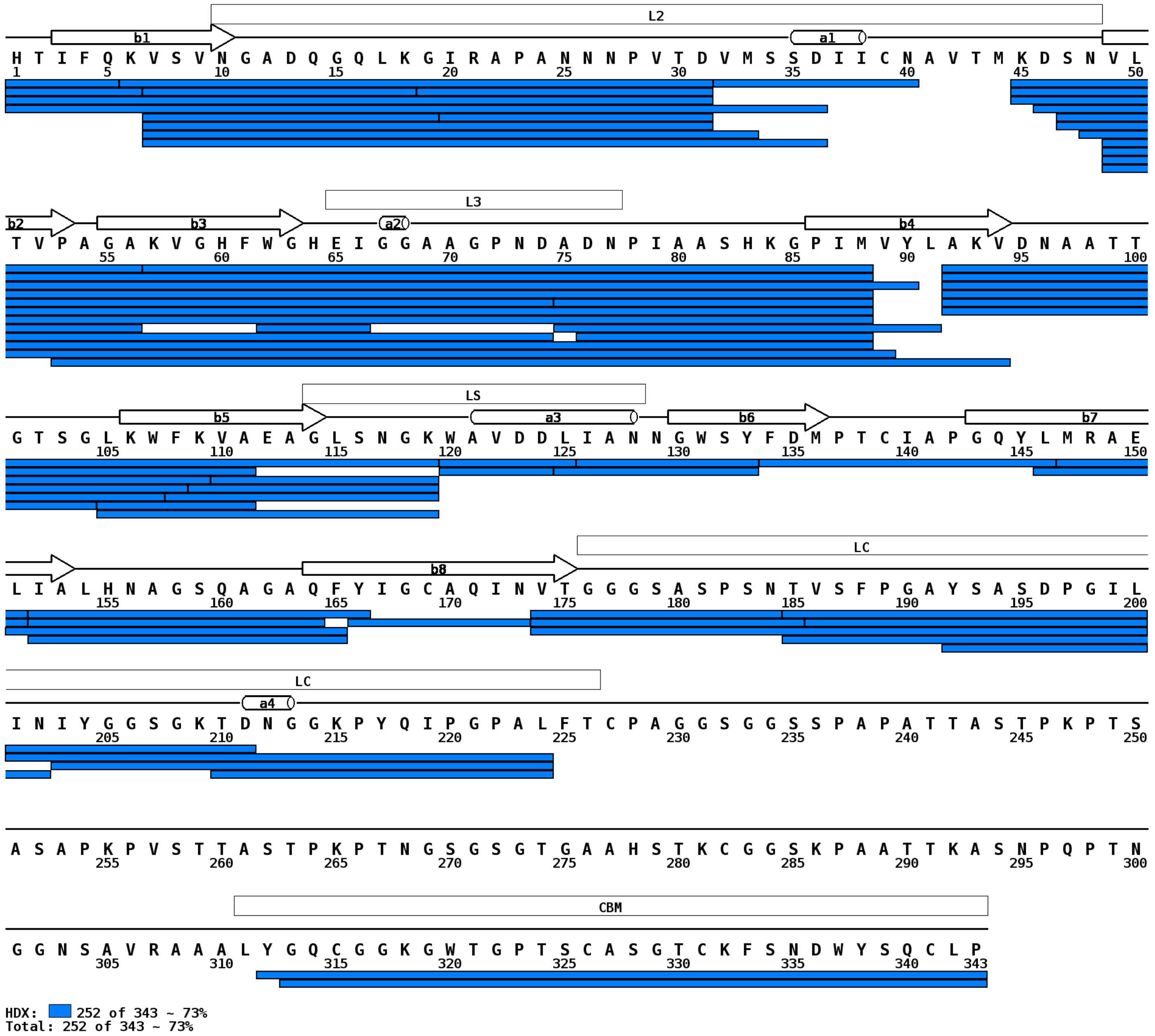

Figure S2. *Nc*LPMO9C structural changes induced by the temperature. Comparison of the *Nc*LPMO9C holo- (top) versus apoenzyme (bottom) at 35 °C (A), 50 °C (B) and 65 °C (C) using mirror plots. Individual time points are shown as rainbow gradient – ***20 sec***, ***2 min***, ***20 min***, ***2 hrs*** and ***6 hrs***. Individual peptides ordered by their "midpoint" value are shown on the X-axis. The Y-axis represents the percentage of deuteration.

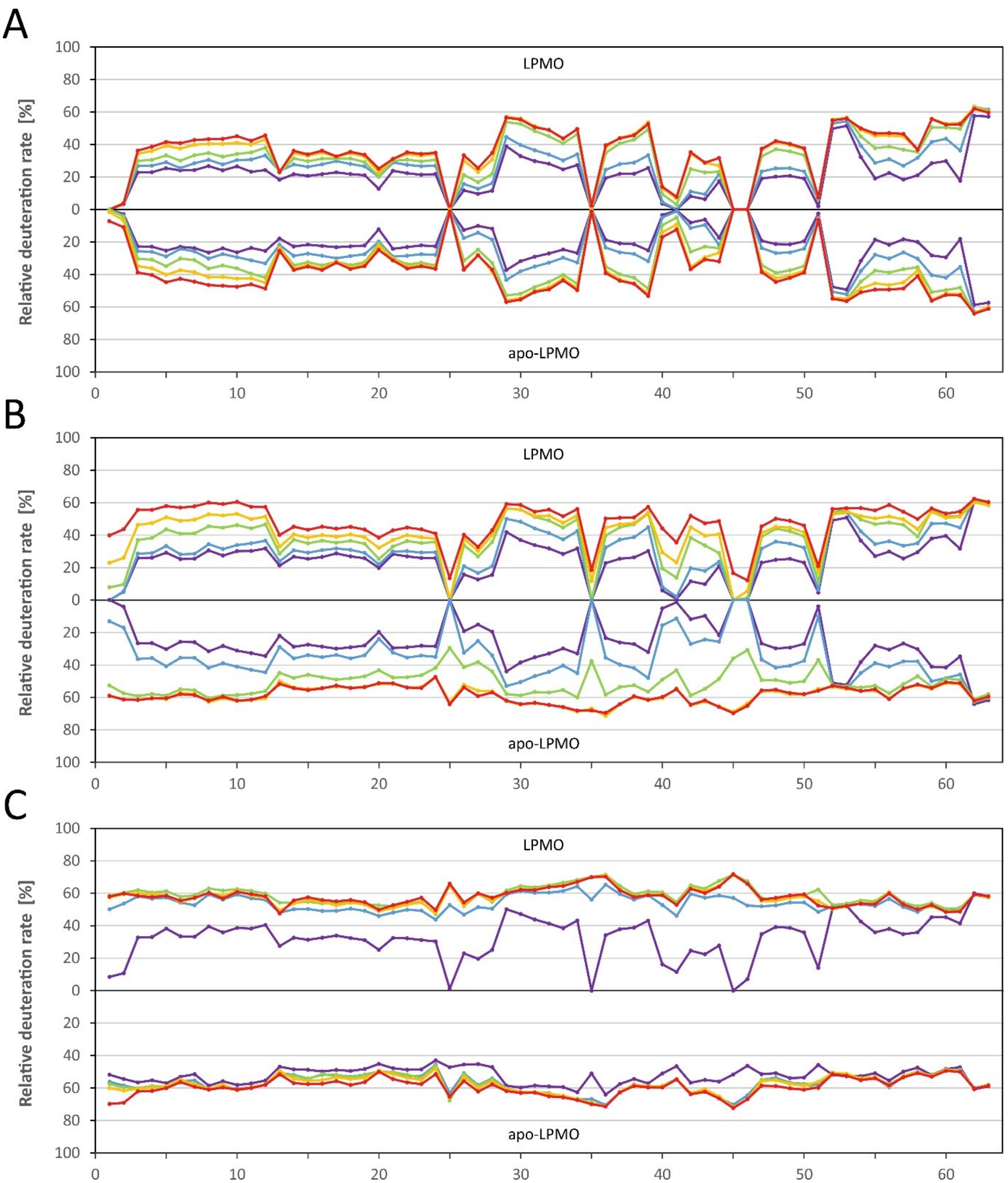

Figure S3. Selected examples of deuterium uptake curves for holo-/apo- *Nc*LPMO9C after temperature correction. *Nc*LPMO9C holo- and apoenzyme were analyzed by HDX-MS at different temperatures (35 °C, 50 °C and 65 °C). Exchange times were 20 ses, 2 min, 20 min, 2 hrs and 6 hrs. The difference in the intrinsic exchange rate was compensated (normalized to 35 °C) by correction factors applied to exchange times. These factors were 3.628 for 50 °C and 11.739 for 65 °C. The individual curves are showing the apo- and holo-LPMO at 35 °C (***grey*** and ***black***, respectively), 50 °C (***light blue*** and ***dark blue***, respectively) and 65 °C (*orange* and ***red***, respectively). The displayed peptides are representative examples of different HDX behaviour across the protein sequence. Peptides are covering regions: Histidine brace (1-6, 75-91, 166-173, indicated with red star symbol), protein structure core (7-18, 20-31, 45-56, 75-91, 120-125, 152-165) as well as the external loops (92-104, 174-184, 201-224) and CBM (313-343).

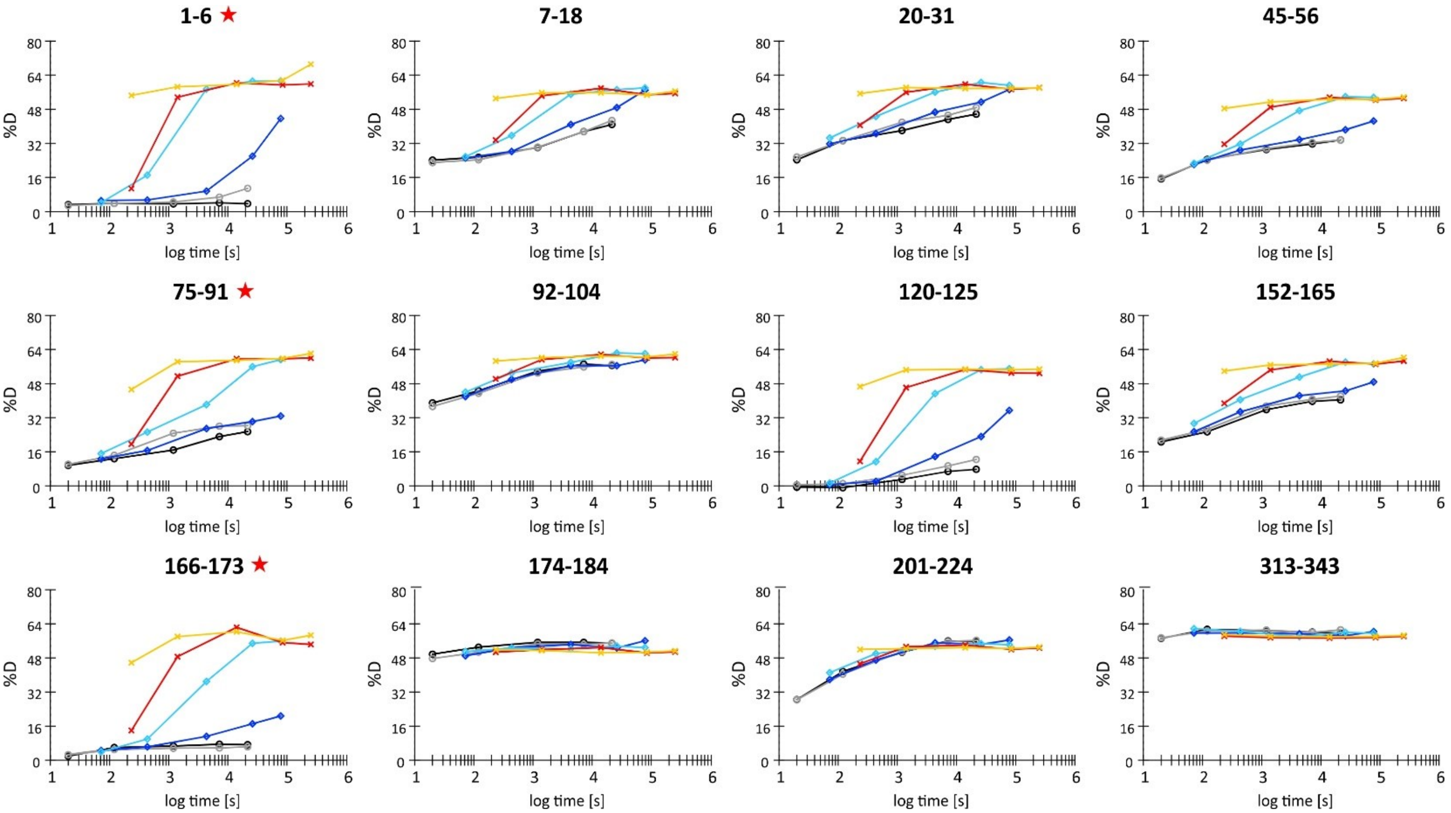

Figure S4. Destabilization of the *Nc*LPMO9C by active site copper removal as observed by HDX-MS comparison of holo- and apoenzymes. Deuteration levels of the holoenzyme were subtracted from those obtained for the apoenzyme. The resulting difference was used to color the *Nc*LPMO9c structure (pdb: 4D7U) by white-red gradient ranging from 0 to 40 %. Data obtained at individual time points of the HDX kinetics (20s, 2m, 20m, 2h, 6h – shown at the top) and different temperatures - 35 °C (A), 50 °C (B) and 65 °C (C) are shown. The copper ion is represented by a green sphere and the side chains of the coordinating residues (His1, His83 and Tyr166) are shown as sticks. A large part of the linker and the CBM are not shown since they are not present in the solved high-resolution structure (4D7U). However, no data were obtained for the linker (residues 225-311) and no difference in deuteration was found on the CBM implying that Cu^{2+} depletion has no detectable effect on this distant domain. The regions not covered by HDX data (41-44) and (225-227) are shown in dark grey.

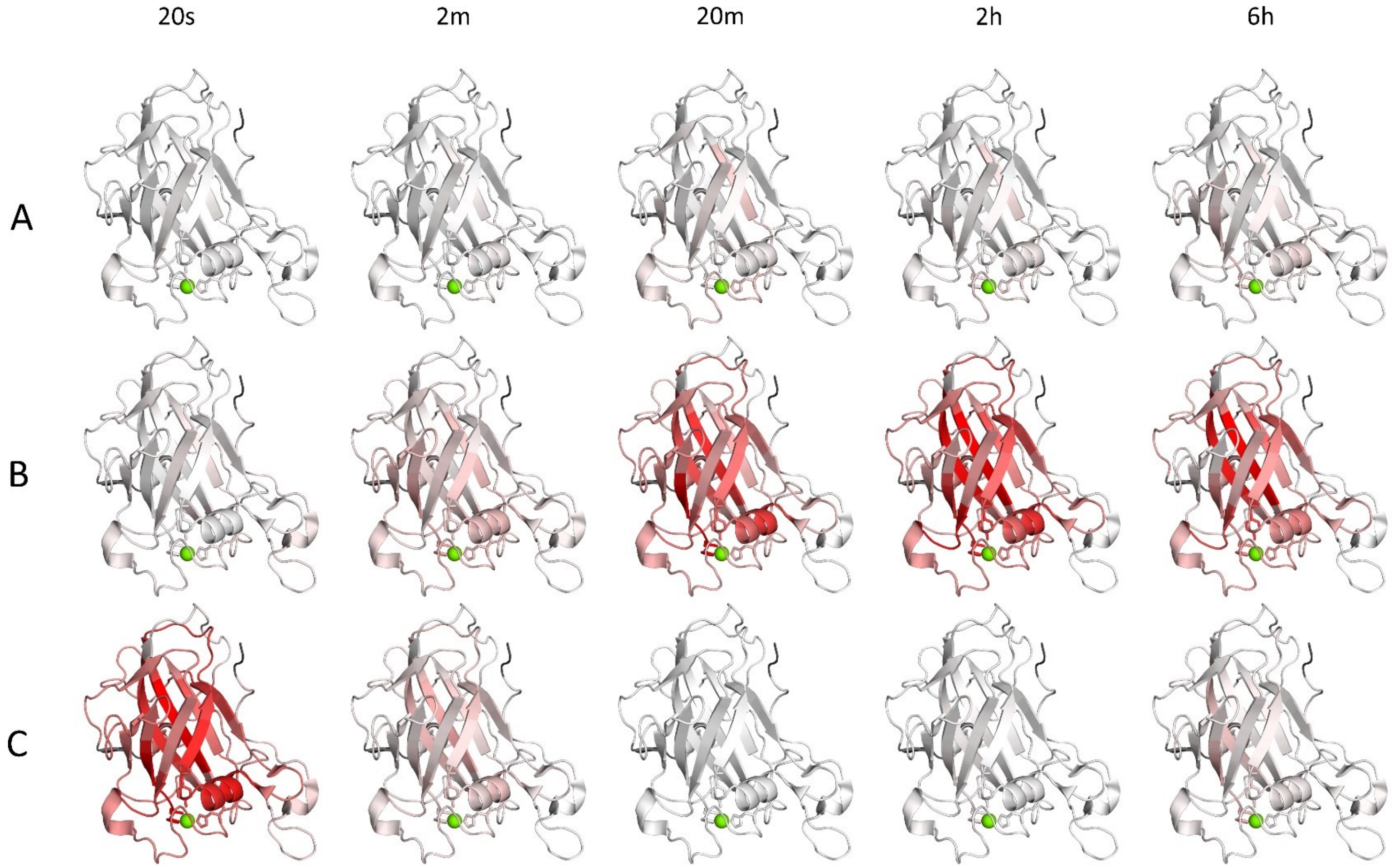

Figure S5. *Nc*LPMO9C structural changes induced by reduction with ascorbic acid at different temperatures. Comparison of oxidized (top) versus ascorbic acid-reduced (bottom) form of *Nc*LPMO9c at 35 °C (A), 50 °C (B) and 65 °C (C) using mirror plots. Individual HDX time points are shown as rainbow gradient – ***20 sec***, ***2 min***, ***20 min***, ***2 hrs*** and ***6 hrs***. Individual peptides ordered by their "midpoint" value are shown on the X-axis. Y-axis represents the percentage of deuteration.

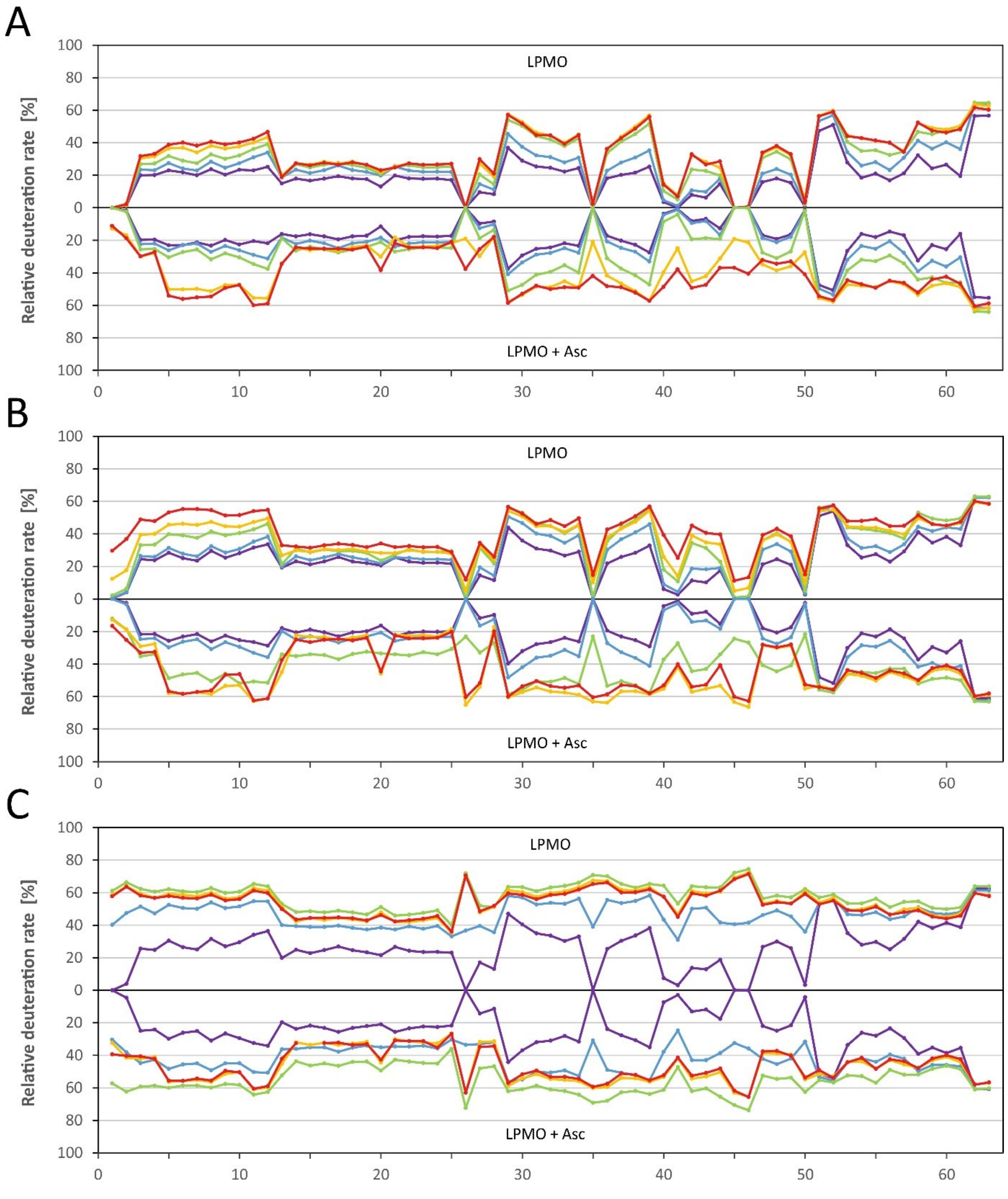

Figure S6. Ascorbic acid reduction causes a range of effects in HDX kinetics and a signal loss. (A) Selected deuterium uptake curves showing oxidized (black) and ascorbic acid-reduced (red) form of *Nc*LPMO9C at 50 °C. There is a detectable lower deuteration of the reduced form in the initial times of exchange which is then reverted to higher deuteration, in some cases followed by a rapid deuteration decrease (an artificial effect caused by oxidation and degradation of the original unmodified peptides). This effect is supported by the plots following signal intensity - Log Int plots - (B) demonstrating signal intensity loss in the reduced samples after longer incubation times. This signal loss corresponds with the position of the peptides around the active center. This can be inferred from the panel (C) where the peptides shown in the graphs were mapped onto the structure of *Nc*LPMO9C. Peptides containing active site histidine brace residues are marked with a red star symbol. Sidechains of amino acids susceptible to oxidation (Met, His, Tyr, Trp, Phe) are shown in the colored regions as sticks. The copper ion is depicted as a green ball. Peptide limits are shown at the top of each graph together with numbers 1-8 which are used to localize the position of each peptide on the structure in panel (C).

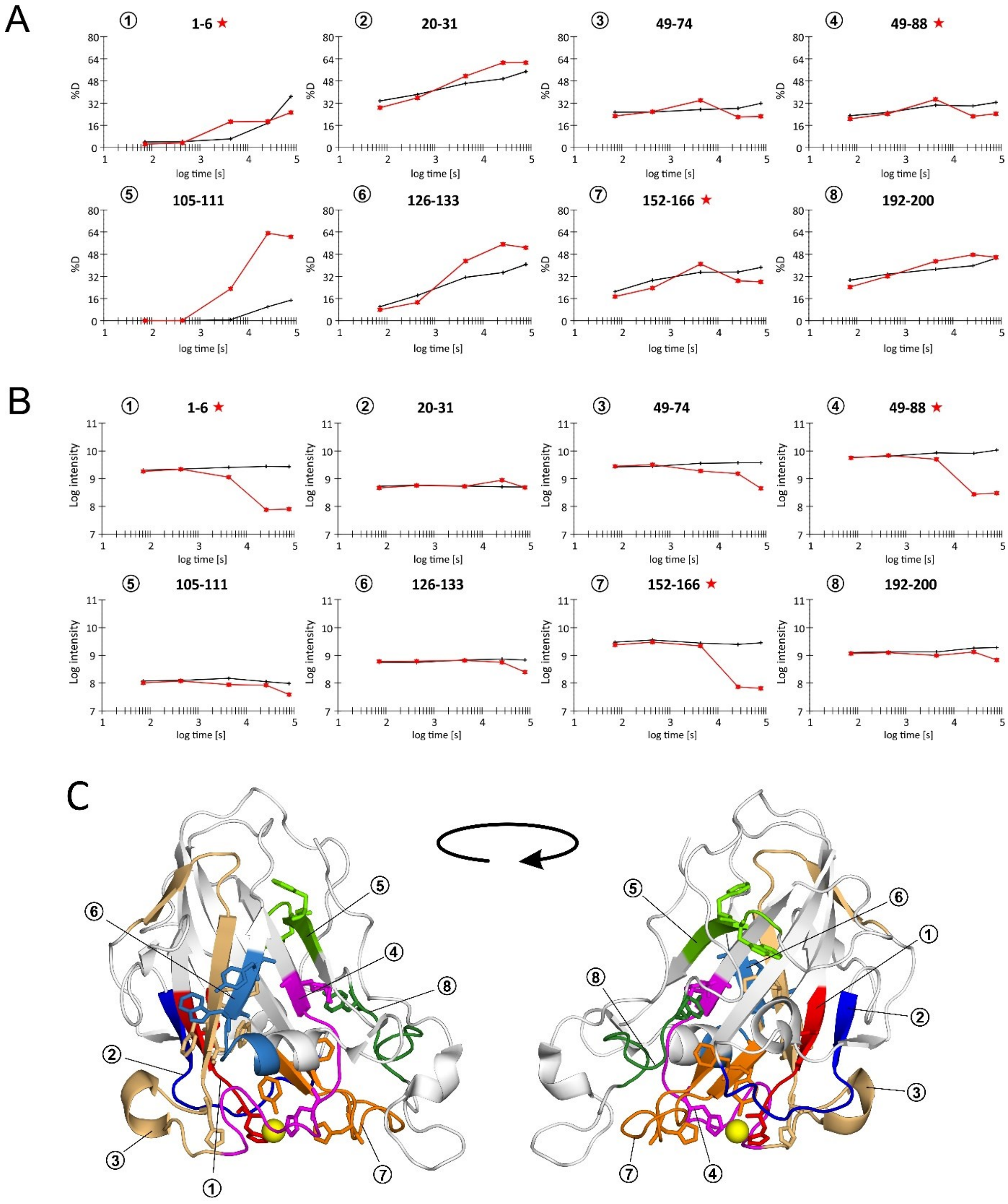

Figure S7. The effect of ascorbic acid-mediated copper ion reduction mapped on the structure of *Nc*LPMO9C. Deuteration levels of the ascorbic acid-reduced form were subtracted from those obtained for the oxidized LPMO and the resulting differences were used to color the *Nc*LPMO9C structure (pdb: 4D7U) by a blue-white-red gradient covering ranges -15 – 0 – 70. Due to very small effects at 35 °C and mixed-effects (reduction-oxidation-damage) at longer time scale at 50 °C and all times at 65 °C, only selected times points (20 sec, 2 min and 30 min) at 50 °C are shown. The copper ion is represented by a green sphere and the side chains of the coordinating residues (His1, His83 and Tyr166) are shown as sticks. Large parts of the linker and the CBM are not shown since they are not present in the crystal structure of *Nc*LPMO9C (pdb: 4D7U). However, no data were obtained for the linker (residues 225-311) and no difference was found on the CBM implying that Cu reduction has no detectable effect on this distant domain. The regions not covered by HDX data (41-44) and (225-227) are shown in dark grey. This figure is a representative example only and a more comprehensive display is provided in the final experiment where optimized reaction/HDX conditions and a shorter sampling interval were used (Figure S9).

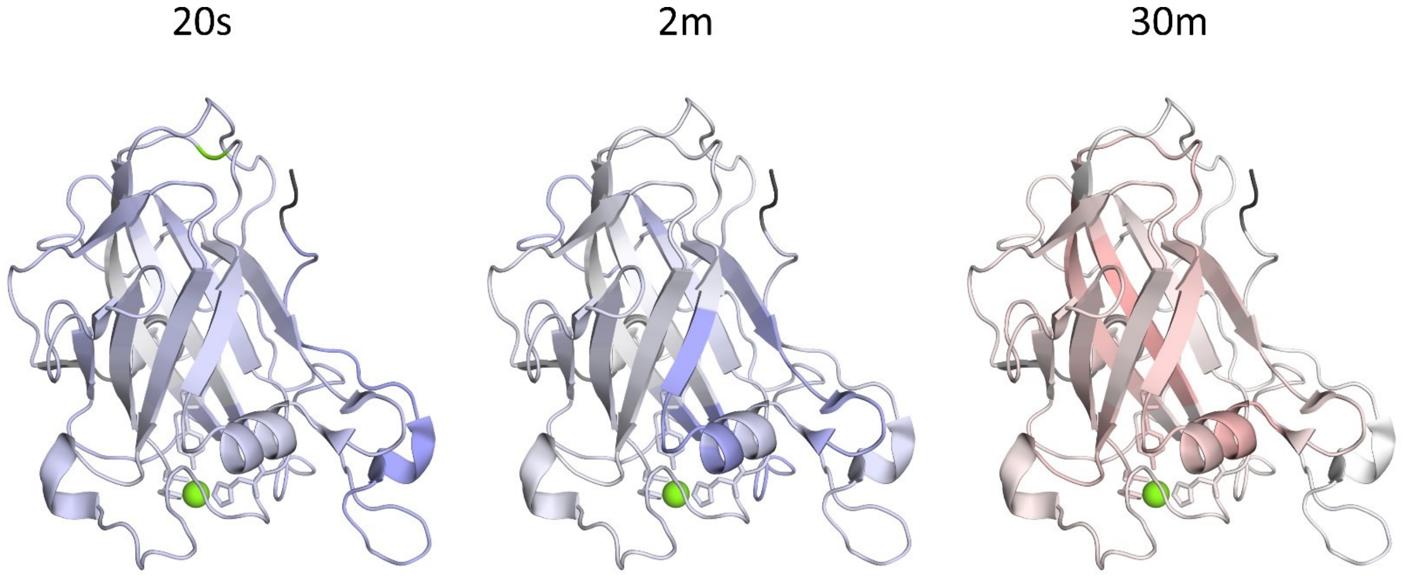

Figure S8. The effects of the reduction of *Nc*LPMO9C by Asc and cellulose-binding in the oxidized and Asc-reduced states. (A) When oxidized *Nc*LPMO9C and cellulose were mixed together, no difference in deuteration was detected as can be judged from the identical HDX profiles of free LPMO (top) and LPMO mixed with microcrystalline cellulose (bottom). (B) Comparison of the oxidized (top) and ascorbic acid-reduced *Nc*LPMO9C (bottom) shows protection at the early times points of the reduced state, which is later reverted to higher deuteration and subsequent degradation due to oxidative damage. (C) Comparison of the reduced *Nc*LPMO9C (top) and reduced LPMO incubated with cellulose (bottom) demonstrates a protective effect of the substrate on the LPMO through slower oxidation and degradation. Experiments were carried out at 50 °C and HDX time points are shown as a rainbow gradient – ***3 min***, ***5 min***, ***10 min***, ***15 min***, ***20 min***, ***25 min***, ***30 min***, ***35 min***, ***40 min*** and ***45 min***. Individual peptides ordered by their "midpoint" value are shown on the X-axis. Y-axis represents the percentage of deuteration.

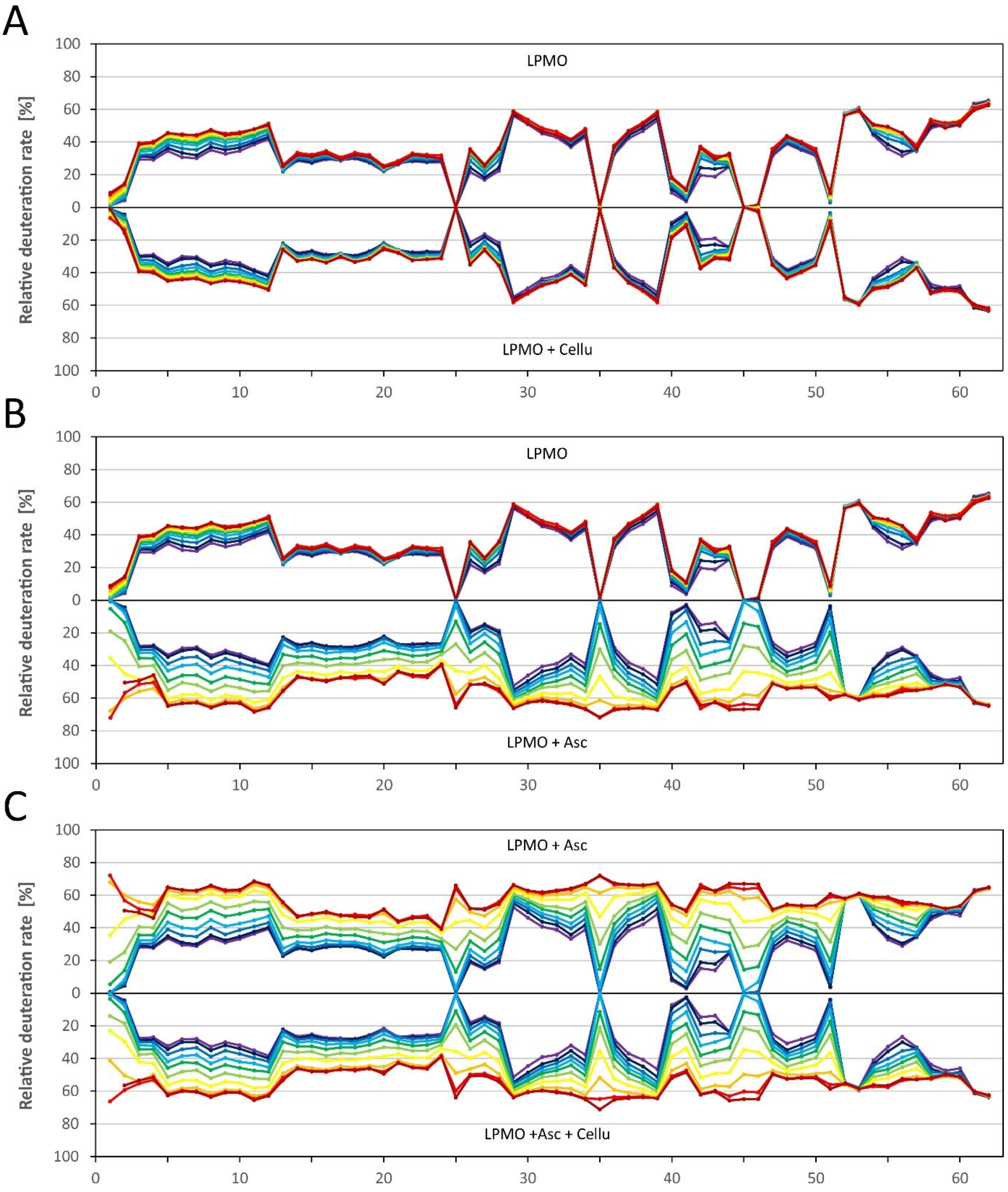

Figure S9. The effects of *Nc*LPMO9C ascorbic acid reduction and cellulose-binding of the Asc-reduced LPMO visualized on the protein structure. (A) The deuteration levels of the ascorbic acid-reduced form were subtracted from those obtained for the oxidized LPMO and the resulting differences were used to color the *Nc*LPMO9C structure (pdb: 4D7U) by blue-white-red gradient covering ranges -15 – 0 – 70, similarly to those shown in **Figure S7**. Individual HDX times are indicated above the structures. Protection caused by ascorbic acid-reduction can be seen for the three initial exchange times (3, 5 and 10 min). Structure opening due to oxidation was detected after 15 min and increases gradually throughout the subsequent incubation time. (B) The effects of cellulose-binding and LPMO protection were detected as a reduction in the extent of oxidative damage and are shown by a white-blue gradient. Similarly to previous cases, deuteration levels of ascorbic acid-reduced LPMO were subtracted from the HDX levels of reduced, cellulose bound LPMO. No effect could be detected at the initial exchange times, indicating that no changes induced by LPMO binding to the substrate can be detected by HDX-MS. On the other hand, slower LPMO oxidation becomes apparent after 15 min of incubation, where oxidation effects became detectable in free LPMO (see panel A). This protection lasted approximately 30 min before fading again. Most likely, the substrate-mediated stabilization was overridden by the massive and fast oxidation/degradation.

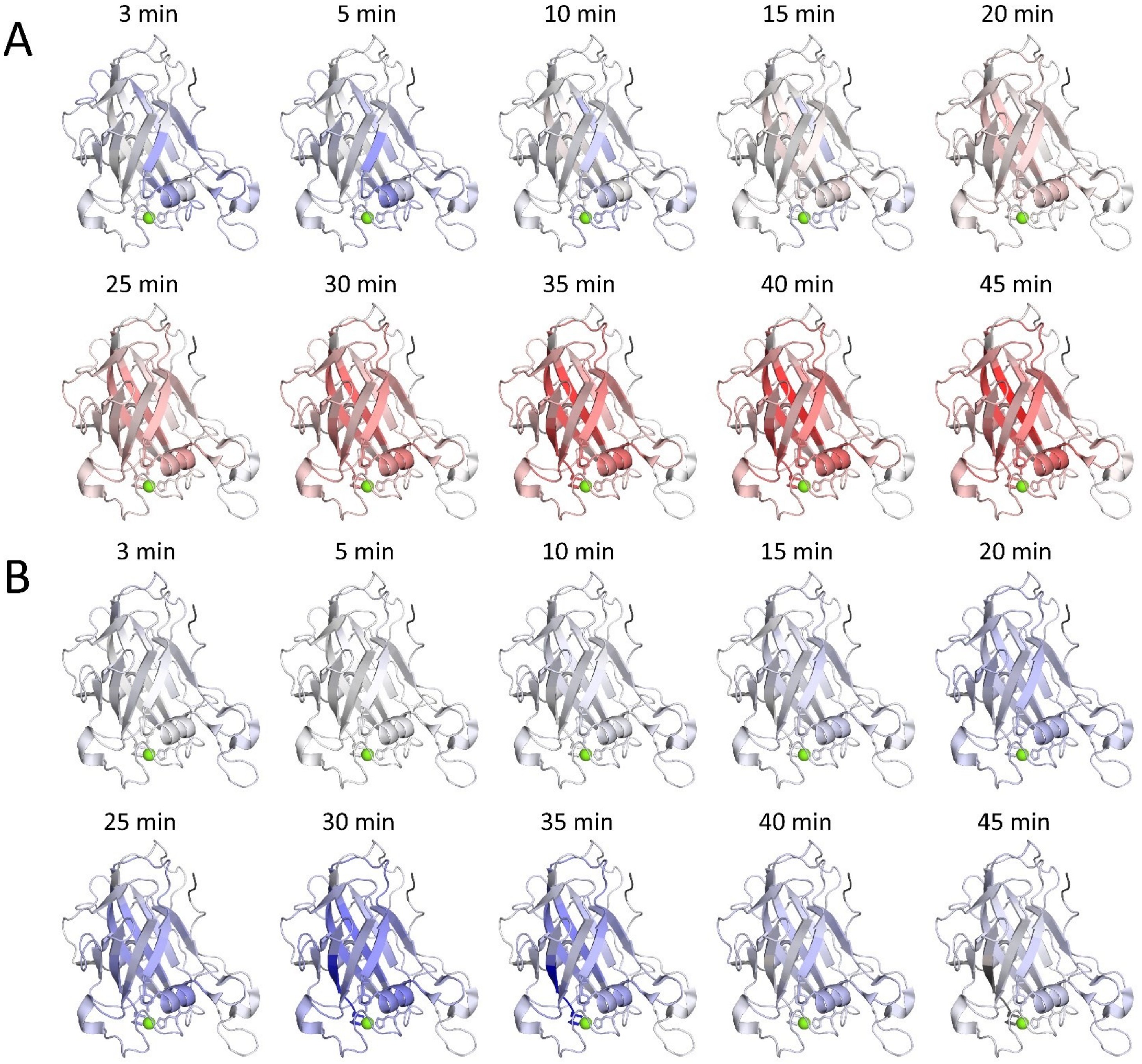

Figure S10. Detailed monitoring of ascorbic acid reduction and its effects on HDX kinetics and signal loss. (A) Selected deuterium uptake curves showing oxidized (**black**), ascorbic acid-reduced (**red**), oxidized in the presence of cellulose (**green**) and reduced in the presence of cellulose (**blue**) form of *Nc*LPMO9C at 50 °C. This experiment is an extended version of the experiment shown in **Figure S6** with the following time points: 3, 5, 10, 15, 20, 25, 30, 35, 40, and 45 min. The data show lower deuteration of the reduced form in the initial times of exchange which is then reverted to higher deuteration and in some cases followed by a rapid deuteration decrease (an artificial effect caused by oxidation and degradation of the original unmodified peptides). This effect is highlighted by the Log Int plots (B) demonstrating signal intensity loss in the reduced samples after longer incubation times. This signal loss corresponds well with the position of the peptides around the active center. This can be inferred from the panel (C) where the peptides shown in the graphs were mapped onto the structure of *Nc*LPMO9c. Peptides containing active site histidine brace residues are marked with a red star symbol. Sidechains of amino acids susceptible to oxidation (Met, His, Tyr, Trp, Phe) are shown in the colored regions as sticks. The copper ion is depicted as a green sphere. Peptide limits are shown at the top of each graph together with number 1-8 which is used to localize the position of each peptide on the structure in panel (C).

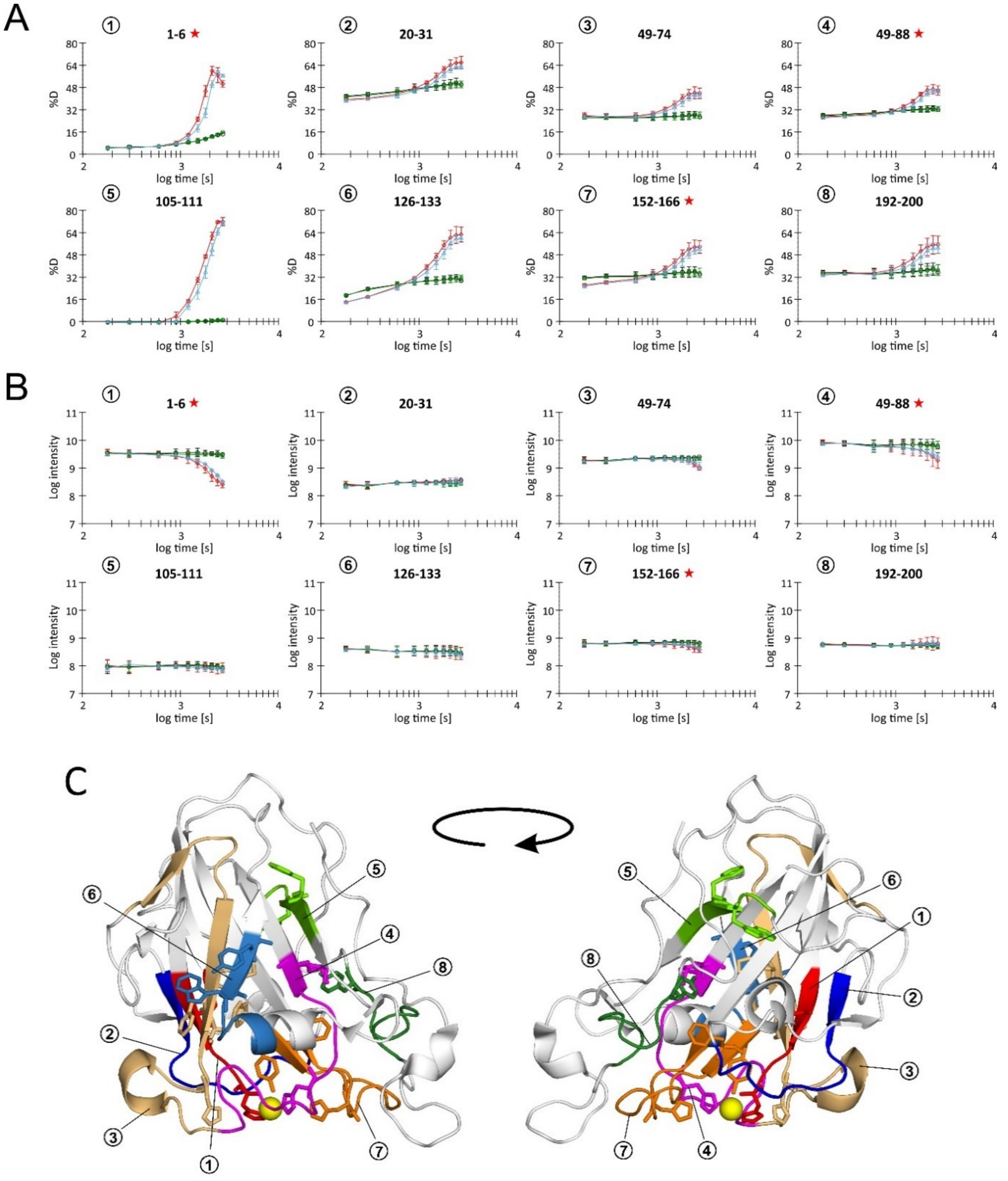

Figure S11. Oxidation induced by copper ion reduction detected in the HDX dataset. Peptide 1-6, covering the His1 of the histidine brace (indicated by a red star symbol) coordinating the active site copper ion becomes increasingly oxidized throughout the experiment. Unoxidized and oxidized forms are showing different HDX kinetics, as can be evidenced from panel (A) where deuterium uptake plots are shown for free (**red**) and cellulose bound (**blue**) and Asc-reduced LPMO. (B) Degradation/oxidation can be deduced from intensity decrease shown in the LogInt plot of unoxidized peptide and inversely in the intensity increase of the oxidized one. It is apparent from all the plots that the presence of cellulose decreased the oxidation rate and structure unfolding and also causes slower degradation of unoxidized peptide and slower intensity increase of the oxidized peptide.

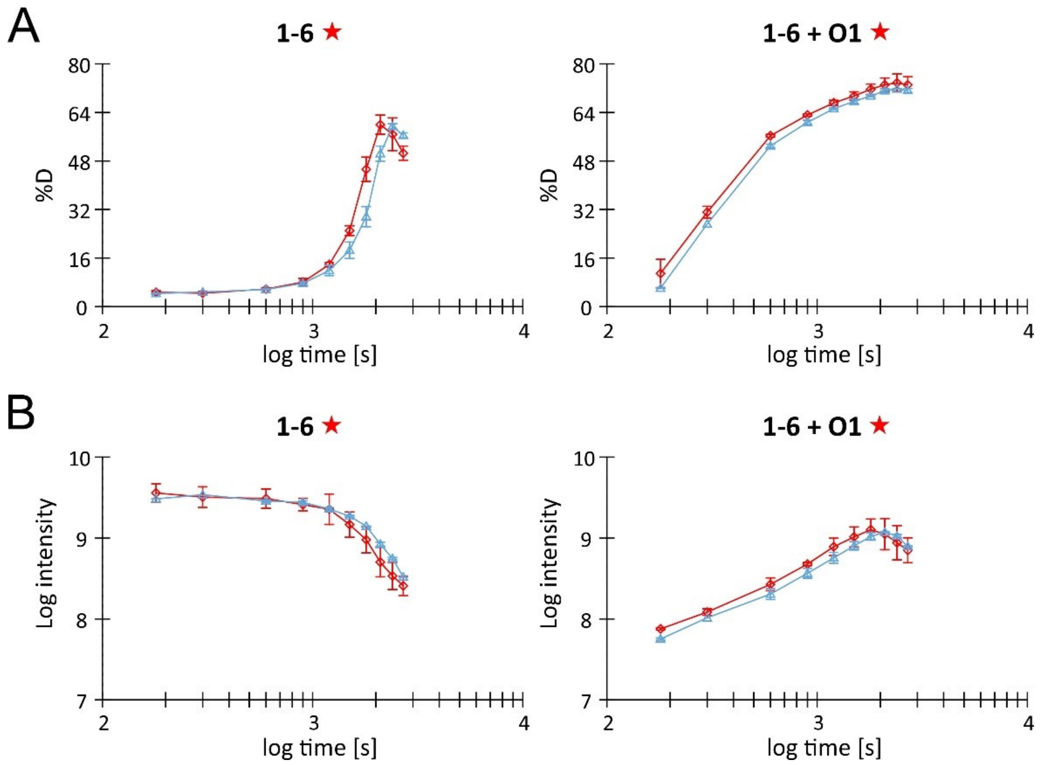

Figure S12. Monitoring of the signal intensity increase of modified peptides. Extracted ion chromatograms for selected singly oxidized (+15,995 Da) peptides obtained by LC-MSMS analysis of samples digested by trypsin. A control sample without ascorbic acid (0 min - **blue**) was compared to samples containing 5 mM Asc incubated for 10 (**green**), 20 (**yellow**) or 30 (**red**) minutes. Residues most susceptible to oxidative modification (Trp, His, Tyr, Met, Cys) on the featured peptides are shown as sticks. Peptides 22-45 and 149-209 show a continuous increase in oxidation while peptide 58-84 captured fast oxidation which was followed by further signal loss linked to oxidation-related events (additional oxidation, cleavage, etc.). Peptide 94-109 represents a peptide with a slow oxidation rate. From the structural point of view, the oxidation rate agrees well with the spatial distribution of these regions on the *Nc*LPMO9C structure as well as with the distribution of oxidizable residues. This can be inferred from the colored structure, where the peptide with the slowest oxidation kinetics is located far from the active site (94-109, **blue**), while faster kinetics can be observed for peptides 22-45 (**orange**), 58-84 (**red**) and 149-209 (**cyan**). The difference between the oxidation rates in peptides 58-84 and 149-209 can most likely be accounted to the involvement of differently positioned active site residues, with His83 being more solvent-exposed than Tyr166, which is positioned behind the copper ion and, at the same time, is closer to the copper ion than His155 which is not involved in the histidine brace motif. Peptide 22-45, while being relatively close to the active site, carries no residue susceptible to oxidation on the loop closest to the copper ion. The relatively high rate of oxidation of this peptide even with oxidizable residues much further away from the active site compared to peptides 58-84 and 149-209, is probably caused by the fact that oxidizable residues in this peptide comprise two methionines and one cysteine. Both amino acids are extremely susceptible to oxidative modification, which probably compensates for their spatial distance.

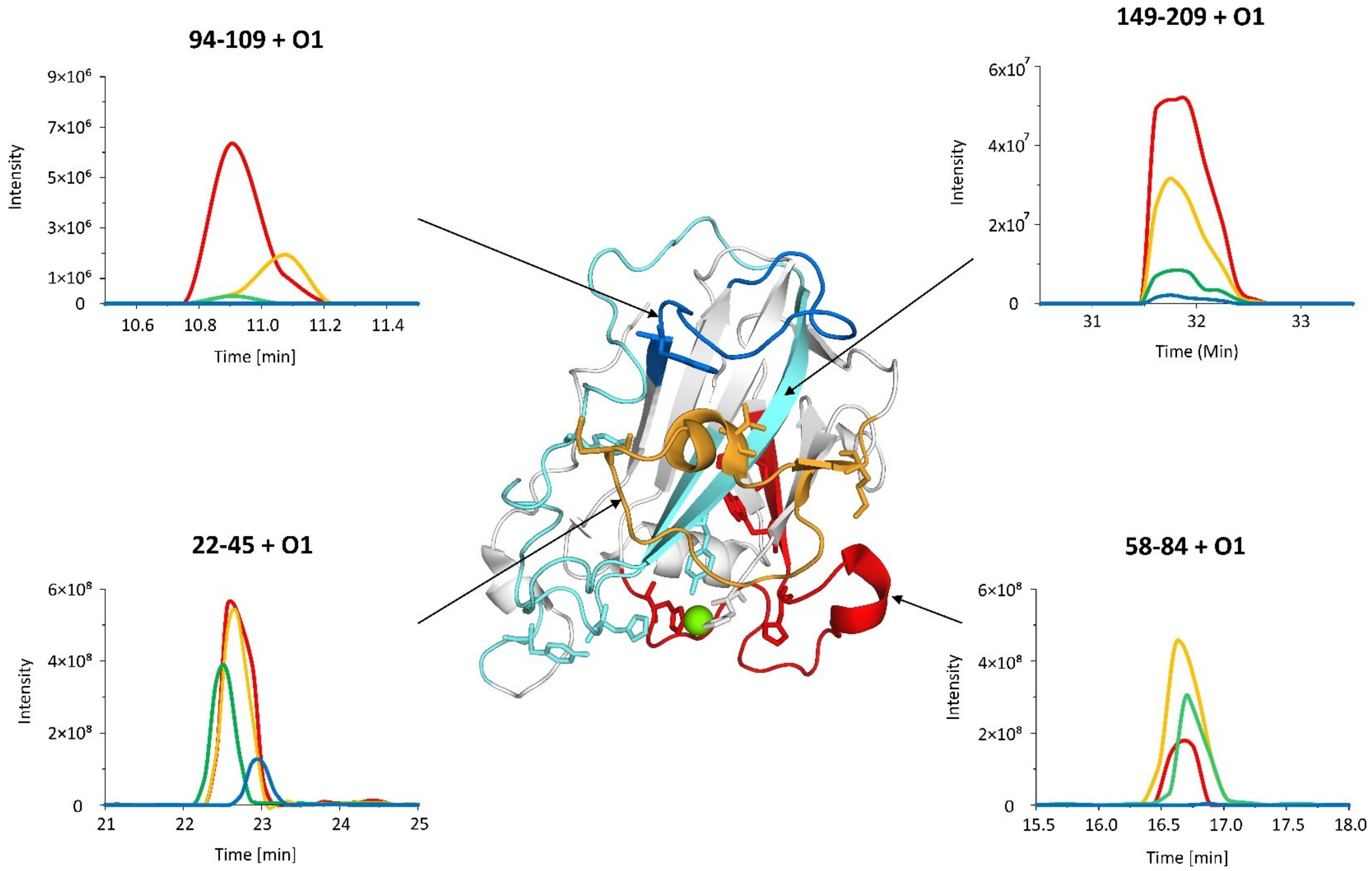

Table S1. Manually verified modifications and cleavages on amino acids in LPMO recorded by MS/MS. The modifications were found in samples collected at various time points (10, 20 and 30 min) containing 5 mM ascorbic acid as LPMO reductant. The positions were pinpointed by observing peptide fragment ions. Positions shown as a range indicate the peptides on which the modification was found and the residue modified is shown in **red**.

Type of modification	Residue	Position	Mass change [Da]	Resulting change
Oxidative peptide cleavage	Thr	**2**-12	+25,979	N-term isocyanate
Oxidative peptide cleavage	Gly	13-**59**	-0,985	C-term amidation
Oxidation	Met	33	+15,995	Methionine sulfoxide
Oxidation	Met	43	+15,995	Methionine sulfoxide
Oxidative peptide cleavage	Val	46-**58**	-0,985	C-term amidation
Oxidative peptide cleavage	Gly	46-**59**	-0,985	C-term amidation
Oxidation	His	60	+15,995	Oxo-Histidine
Oxidative breakdown	His	60	-22,03	Aspartate
Oxidation	Trp	62	+15,995	Hydroxyl-Trp
Oxidation	His	64	+15,995	Oxo-Histidine
Oxidative breakdown	His	64	-22,03	Aspartate
Oxidation	His	83	+15,995	Oxo-Histidine
Oxidation	Met	88	+15,995	Methionine sulfoxide
Oxidation	Trp	107	+15,995	Hydroxyl-Trp
Oxidation	Trp	131	+15,995	Hydroxyl-Trp
Oxidation	Tyr	133	+15,995	Hydroxyl-Tyr
Oxidation	Met	147	+15,995	Methionine sulfoxide
